# Wh-filler-gap dependency formation guides reflexive antecedent search

**DOI:** 10.3389/fpsyg.2015.01504

**Published:** 2015-10-09

**Authors:** Michael Frazier, Lauren Ackerman, Peter Baumann, David Potter, Masaya Yoshida

**Affiliations:** Department of Linguistics, Northwestern UniversityEvanston, IL, USA

**Keywords:** reflexive antecedent search, filler-gap dependency resolution, structure-sensitivity, gender mismatch effect, eye-tracking, text-reading

## Abstract

Prior studies on online sentence processing have shown that the parser can resolve non-local dependencies rapidly and accurately. This study investigates the interaction between the processing of two such non-local dependencies: *wh*-filler-gap dependencies (WhFGD) and reflexive-antecedent dependencies. We show that reflexive-antecedent dependency resolution is sensitive to the presence of a WhFGD, and argue that the filler-gap dependency established by WhFGD resolution is selected online as the antecedent of a reflexive dependency. We investigate the processing of constructions like (1), where two NPs might be possible antecedents for the reflexive, namely *which cowgirl* and *Mary*. Even though *Mary* is linearly closer to the reflexive, the only grammatically licit antecedent for the reflexive is the more distant *wh*-NP, *which cowgirl*.

*(1). Which cowgirl did Mary expect to have injured herself due to negligence*?

Four eye-tracking text-reading experiments were conducted on examples like (1), differing in whether the embedded clause was non-finite (1 and 3) or finite (2 and 4), and in whether the tail of the *wh*-dependency intervened between the reflexive and its closest overt antecedent (1 and 2) or the *wh*-dependency was associated with a position earlier in the sentence (3 and 4). The results of Experiments 1 and 2 indicate the parser accesses the result of WhFGD formation during reflexive antecedent search. The resolution of a wh-dependency alters the representation that reflexive antecedent search operates over, allowing the grammatical but linearly distant antecedent to be accessed rapidly. In the absence of a long-distance WhFGD (Experiments 3 and 4), *wh*-NPs were not found to impact reading times of the reflexive, indicating that the parser's ability to select distant *wh*-NPs as reflexive antecedents crucially involves syntactic structure.

## 1. Introduction

In order to interpret sentences of natural language, the human parser must establish non-local dependencies between elements received in the input. Two such kinds of dependencies are wh-dependencies and reflexive-antecedent dependencies. The former is the dependency between a *wh*-word such as *who* or *which* and the empty argument position (e.g., subject, direct object, indirect object) where it is interpreted, which we refer to throughout as a “*Wh*-filler-gap dependency” or “WhFGD.” The latter is the dependency between a reflexive pronoun such as *himself* or *herself* and the antecedent noun phrase on which it is referentially dependent, which we refer to as a “reflexive dependency” or “RD.”

WhFGDs and RDs differ from one another in a number of ways. While in a WhFGD the presence of a *wh*-word at the left edge of a clause can provide evidence for the existence of an empty NP position later on, such as the empty direct object position in a sentence like *What did Mary eat?*, a RD cannot be recognized until later. This is because in a RD, it is typically the later-occurring element in the dependency that contains bottom-up evidence of the need for a reflexive-antecedent relation. That is, in a sentence like *Mary saw herself*, there is no indication that *Mary* will need to be associated with a reflexive later in the sentence until the reflexive *herself* is actually encountered. Evidence from many psycholinguistic studies, discussed below, indicates that both of these dependency resolution processes occur quite rapidly in online reading. If the presence of a WhFGD affects the online operation of a subsequent RD resolution, this would constitute evidence that the antecedent retrieval process is sensitive to syntactic structure, namely to the presence and location of the WhFGD.

In this paper, we investigate the interaction between the processes of the parser that establish these two kinds of dependencies in on-line sentence comprehension. In particular, we examine whether resolving a WhFGD establishes a new candidate antecedent in the representation that is searched during the resolution of a RD. Converging evidence from the psycholinguistic sentence processing literature indicates that the process of WhFGD resolution is an active process. In particular, upon encountering a *wh*-word, the parser does not wait to receive bottom-up information determining the location of the tail of the WhFGD, but actively posits or hypothesizes the dependency tail whenever it detects an incoming position at which resolving the dependency would be grammatically licit (Stowe, 1986; Traxler and Pickering, 1996; Phillips, 2006). Likewise, reflexive resolution is known to be rapid and grammatically sensitive (Nicol and Swinney, 1989; Sturt, 2003; Jäger et al., 2015): upon encountering the reflexive, the parser tries to link the reflexive to grammatically licit antecedents in the early stages of online processing.

Considering RDs like 1, a reflexive normally co-refers with its closest potential antecedent. In (1), *himself* is understood to co-refer with *the man*, not with *Jane*^1^. In (2), however, the *wh*-phrase *which man* is interpreted as the subject of the non-finite embedded clause *to have injured himself*, just as *the man* is in (1), but it is displaced from the canonical embedded subject position after *expect*. In a context such as this, the *wh*-phrase *which man* must be the antecedent of *himself*, instead of the linearly closer noun phrase *Jane*. If *Jane* were chosen as the antecedent of the reflexive in either (1) or (2), the example would be predicted to be unacceptable due to the gender mismatch between the feminine name *Jane* and the masculine reflexive *himself*, contrary to fact.

(1) Jane_*i*_ expected the man_*j*_ to have injured himself_^*^i∕*j*_.
(2) Which man_*i*_ did Jane_*j*_ expect to have injured himself_*i*∕^*^j_?

Examples such as these, with nonfinite embedded clauses associated with sentence-initial *wh*-phrases, allow us to investigate whether the result of WhFGD resolution influences RD resolution. Without WhFGD resolution, in an example like (2) the closest potential candidate antecedent (*Jane*) for the reflexive mismatches with it in gender. If reflexive resolution operates over a representation that does not include information about WhFGDs, then in the course of finding the antecedent for the reflexive *himself* in (2) the parser may (at least transiently) attempt to associate *himself* with *Jane*, leading to processing difficulty and a possible slowdown due to the gender mismatch (Sturt, 2003).

If, however, the active process of WhFGD resolution alters the representation over which reflexive resolution operates, it may establish a new candidate antecedent for the reflexive that is closer to the reflexive than the ungrammatical antecedent *Jane*, by co-indexing the sentence-initial *wh*-phrase *which man* with the position of the gap (3)^2^. In this case, the closest candidate antecedent for the reflexive in (2) will be the gap linked to the (masculine) *wh*-phrase. We would thus not expect the parser to attempt to associate *himself* with *Jane* even temporarily, because there is a closer, grammatically acceptable antecedent. The parser should therefore experience no gender-mismatch effect when the reflexive mismatches in gender with an ungrammatical but linearly close candidate antecedent such as *Jane* in (2).

(3) Which man_*i*_ did Jane expect /gap/_*i*_ to have injured himself?

Thus, whether or not the parser experiences gender-mismatch effects from a linearly close but ungrammatical candidate antecedent like *Jane* in examples like (2) can tell us whether the process of reflexive resolution is sensitive to the presence of a WhFGD.

The plan for this paper is as follows. In the remainder of Section 1, we discuss the theoretical and empirical background of this line of research, focusing in turn on WhFGD resolution (Section 1.1), and reflexive antecedent search (Section 1.2). In Section 2 we report the results of four experiments to test whether the tail of a WhFGD is treated online as a potential antecedent for reflexive resolution. Section 3 discusses the implications of these results for theories of sentence-processing, and Section 4 concludes.

### 1.1. Active *wh*-dependency resolution

The term *WhFGD resolution* refers to the process by which the parser interprets a left-peripheral *wh*-question element such as *what, who*, or *which NP* to correspond to appropriate sentence-internal material—approximately, to correspond to the position in which the *wh*-element's correlate would appear in an answer to the *wh*-question. The end result of this process is that the *wh*-element in a *wh*-question like (4-a) is interpreted as corresponding to the empty direct object position, such that an answer to (4-a) would include an element filling this position, as in (4-b).

(4)
  a. What did Mary devour?
  b. Mary devoured fish.

Upon encountering a position in the input string in which a grammatically obligatory element is missing, [in the case of (4-a), the object position immediately after *devour*] the parser has bottom-up evidence that this is the position to be affiliated with the left-peripheral *wh*-word. In what follows, we refer to this empty position in the input corresponding to the *wh*-element's answer and to the variable in the sentence's interpretation as the *gap*.

Converging evidence from the psycholinguistic sentence processing literature, however, indicates that the parser is not as conservative in resolving WhFGDs as the above would suggest: instead the process of *wh*-dependency resolution is an *active* process (Frazier, 1987). In particular, upon encountering a *wh*-word, the parser does not wait to receive bottom-up information determining the location of the gap (viz. a position in the input string in which a grammatically obligatory element is missing), but actively posits or hypothesizes the existence of a gap whenever it detects an incoming position at which such a gap would be grammatically licit (Traxler and Pickering, 1996; Phillips, 2006; Omaki et al., 2015).

The principle line of evidence that *wh*-dependency resolution is an active process in this sense comes from the so-called filled-gap effect (FGE, Stowe, 1986 *et seq*.). The FGE is a reading-time slowdown observed at the positions of an overt NP in a sentence with a *wh*-element, such as the position of *the sushi* in (5). The object position after *eat* is a potential gap site, but not an actual gap site, the actual gap site being in the complement of the preposition *with*. The fact that reading-time slowdowns are observed at such positions is interpreted as an indication of the parser's having hypothesized a gap in the position occupied by the overt NP and subsequently, upon finding this prediction falsified, having to take time to correct its mistake^3^.

(5) What did Mary eat the sushi with?

Precisely how active or predictive the process of WhFGD resolution may be is not directly relevant to the present study, because in all experiments reported here the WhFGD occurs substantially before the measurement regions of the reflexive and its spillover region. However, the general finding that gap-filling is an active, rapid process is strong evidence that this process will have completed by the time RD resolution is triggered, when the parser encounters the reflexive in examples like (3). This enables us to study whether reflexive resolution is sensitive to the presence of a WhFGD without the danger that the WhFGD has not been recognized by the parser at the point when RD resolution occurs.

### 1.2. Antecedent retrieval

Because *wh*-words in English are under normal circumstances located at the left edge of the clause with which they are associated, *wh*-dependency resolution is in the general case a forward process in the sense that the cue to the existence of a long-distance dependency between two linguistic elements is encountered at the leftmost element. Reflexive antecedent search is quite different, because while a reflexive is overtly marked with the morpheme *self*, it generally occurs after its antecedent, which does not bear any marking indicating that it is the antecedent of an upcoming reflexive. Trivially, in example (6) below, *John* occurs in the same form whether it is the antecedent of a reflexive (6-b) or not (6-a). That is, while the presence of *wh*-morphology triggers an active search through subsequently-processed linguistic material for the tail of the *wh*-dependency, the presence of reflexive morphology (English *-self*) must instead trigger a backwards search through previously-processed material for its antecedent.

(6)
  a. John_*i*_ dislikes him_^*^i∕*j*_.
  b. John_*i*_ dislikes himself_*i*∕^*^j_.

Additionally, the possibility of a RD is constrained in two ways. The first constraint, typically referred to as Condition A of the Binding Theory (Chomsky, 1981), states that reflexives must be locally bound, so non-clausemate NPs and those that do not c-command^4^ the reflexive are illicit antecedents, as indicated by the unacceptability of the examples in (7). Second, in English, a reflexive must match its antecedent in number and gender, so that e.g., masculine NPs are illicit antecedents of feminine reflexives and vice versa, as indicated by the unacceptability of the examples in (8).

(7)
  a. ^*^John hopes that the police won't find himself.
  b. ^*^Rumors about John bothered himself.

(8)
  a. ^*^John injured herself.
  b. ^*^Mary injured himself.

Although the aim of this study is principally to determine how structure-sensitive the reflexive antecedent retrieval process is, rather than to distinguish between different mechanisms of antecedent retrieval, the parser's behavior in this context still has the potential to be informative about the retrieval mechanism itself, and so some discussion of different models of the retrieval of linguistic antecedents from memory bears inclusion here.

In cue-based models of antecedent retrieval like Lewis and Vasishth (2005), the antecedent retrieval mechanism is not crucially constrained by syntactic structure. Instead, upon encountering a word that requires an antecedent (in the present case, the reflexive), the parser performs a feature-matching operation in parallel on all the elements in a content-addressable memory store—roughly, all the words it has recently encountered. In a model like this, cues indexing the syntactic position of potential antecedents can interact with non-structural cues like agreement features, allowing ungrammatical antecedents to potentially be retrieved. On such an account, both candidate reflexive antecedents are predicted to impact the reading-time measures of the reflexive in our experiments, because both of them will be simultaneously checked against the features (in particular the gender feature) of the reflexive when the parser encounters it.

Precisely how the candidate antecedents should affect reading-time measures of the reflexive depends upon the details of the cue-based model adopted. A simple possibility is that the parser should experience extra difficulty when no candidate antecedent is found in its memory store, leading to an interaction effect such that the reflexive regions of sentences like (3) but containing no gender-matching antecedent for the reflexive, such as (9), are read most slowly.

(9) Which woman did Jane expect to have injured himself?

More complex patterns are also possible, however. In the model of Lewis and Vasishth (2005), two distinct interference effects are predicted between the match/mismatch of the *wh*-NP and the linearly local NP. First, when both candidate antecedents match the feature specification of the reflexive, similarity-based interference is predicted, such that the reflexive regions of sentences like (3) but containing two gender-matching antecedent for the reflexive, such as (10), will exhibit reading-time slowdowns.

(10) Which man did John expect to have injured himself?

This is due to the mutual inhibition between the featurally similar candidates. Second, facilitation should occur where the accessible antecedent mismatches and the inaccessible antecedent matches the features of the reflexive. This would manifest as faster reading times. More complex models such as the one in Jäger et al. (2015) also predict that the gender congruency of the candidate antecedents should interact in modulating reading-times at the reflexive. In general, cue-based retrieval models that are not constrained by syntactic structure make the prediction that both candidate antecedents should affect reading times.

Cue-based models commonly incorporate a decay parameter, such that items that have been in memory longer are less salient and harder to retrieve, but a decay parameter does not predict effects of the *wh*-NP in the absence of effects of the more local candidate antecedent NP, since the *wh*-NP will have been in memory slightly longer. Even if the *wh*-NP is re-activated (and thus boosted) in memory at the position of the verb in the lower clause as Lewis and Vasishth (2005)'s model predicts, it should still be the case that the activation of the more local candidate antecedent remains strong enough to induce some effect at the reflexive. For this reason, theories of cue-based retrieval would predict an interference effect from the matrix subject NP *Jane* in (3).

Dillon et al. (2013) did not observe an interference effect of this kind in their experiments on sentences like (11), and performed computational simulations of the level of memory activation of the competing reflexive antecedents in order to determine whether such a reactivation-based account could explain the lack of interference effects. They compared the predictions of a cue-based system that was restricted to consider only syntactic information in reflexive antecedent retrieval with one that could consider all cues, including agreement information, where both systems incorporated memory reactivation of the grammatical antecedent [in (11), *the new executive*]) at the matrix verb (*doubted*), a point after the competing antecedent (*the middle managers*).

(11) ^*^The new executive who oversaw the middle managers apparently doubted themselves …

They concluded, however, that a formal model of antecedent activation that was restricted to consider only syntactic cues in reflexive antecedent search provided a closer fit to their empirical findings than one that considered all cues (including morphological ones) and depended only upon relative activation level to modulate which candidate antecedent was retrieved. That is to say, cue-based models that were not restricted to consider only syntactic structural cues in reflexive antecedent retrieval predicted more interference than observed, even after accounting for reactivation of the grammatical antecedent.

While the sentences studied in Dillon et al. (2013) involve somewhat different long-distance dependencies, in both their sentences and ours the grammatical antecedent is reactivated after the ungrammatical candidate antecedent [at *doubted* in their (11) and at or around *have injured* in our (3)], and so a model such as theirs plausibly predicts no effect of the ungrammatical candidate antecedent in our experiments as well.

Many previous studies have investigated whether the antecedent retrieval process, whether cue-based or otherwise, is constrained by syntactic relations: namely, where a potential antecedent is located in the syntactic tree (e.g., Badecker and Straub, 2002; Sturt, 2003; Felser et al., 2009; Xiang et al., 2009; Cunnings and Felser, 2013; Dillon et al., 2013; Clackson and Heyer, 2014; Cunnings and Sturt, 2014).

Sturt (2003) investigated the on-line application of Condition A of the Binding Theory in sentence processing by cross-manipulating the (stereotypical) gender match/mismatch and structural accessibility/inaccessibility (in terms of c-command relations) of prior discourse referents. In his Experiment 1, two candidate antecedents, both c-commanding a reflexive, were cross-varied for gender congruency with the reflexive, as in (12). As expected, when the linearly closer and structurally accessible antecedent mismatched the anaphor in stereotypical gender, reading times on the anaphor/spill-over region were slower.

(12) He/she remembered that the surgeon had pricked himself/herself with a used syringe needle.

On the other hand, in Sturt (2003)'s Experiment 1, a significant effect of inaccessible-match/mismatch was found in later measures, such that mismatching inaccessible antecedents slowed down subsequent reading times on the anaphor. Sturt (2003) interpreted this result as evidence that the antecedent retrieval process is structurally constrained such that grammatical constraints act as a filter on interpretation during on-line reading, which can subsequently be violated by more general comprehension processes. Cunnings et al. (2015) and Kush et al. (2015) found similar patterns in the case of pronoun binding, and both interpret them as evidence of later comprehension processes attempting to coerce an antecedence relation when none is permitted by the grammar, though the explanation offered by Kush et al. (2015) involves a number of additional complications.

Sturt (2003)'s Experiment 2 is similar in some ways to the present study in that it also tests reflexive resolution in configurations where a grammatically inaccessible antecedent is linearly closer to the reflexive than the grammatically accessible antecedent.

(13) The surgeon who treated Jonathan/Jennifer had pricked himself/herself with a used syringe needle.

The fact that Sturt found no result of the inaccessible antecedent in his Experiment 2, in contrast with his Experiment 1 where late interference effects were found, may indicate that such interference effects are confined to configurations in which the inaccessible antecedent c-commands the reflexive. If this is the case, interference effects similar to those in Sturt's Experiment 1 may be found in the present study. However, in this study, unlike in Sturt's Experiment 2, both the accessible and inaccessible antecedent c-command the reflexive. This difference allows the present study to serve as a kind of follow-up to Sturt (2003), distinguishing whether the parser's reflexive antecedent resolution system is sensitive to structural (rather than linear) locality of a potential antecedent separately from c-command.

Substantial additional evidence indicates that at least the structural relation of c-command affects dependency formation. Cunnings et al. (2015) and Kush et al. (2015), for example, both investigated the retrieval of antecedents of bound pronouns which, like reflexives, are referentially dependent upon other NPs. Both groups of researchers found evidence that the c-command relation constrains the antecedent search process, such that bound pronouns only trigger antecedent retrieval of possible binders in c-commanding positions.

However, theories taking account of only c-command do not predict that the parser should be able to effectively distinguish between the grammatical and ungrammatical antecedents in our experiments, since both candidate antecedents c-command the reflexive. For these accounts to make different predictions about these sentences, a notion of locality is needed as well—reflexives in English are more restricted than bound pronouns because their antecedents must c-command the reflexive *and* must be contained in the same immediate clause. If the retrieval system can take advantage of both of these structural properties (c-command and clausemate-hood), it should fail to exhibit interference effects from the linearly more local candidate antecedent in sentences like (3).

That is to say, the possible grammatical sensitivity of the parser investigated here is somewhat finer grained than that investigated in e.g., Cunnings et al. (2015), who investigated whether antecedent retrieval is sensitive to the c-command constraint on anaphora. Correctly resolving sentences like (3) requires the parser to attend to two grammatical constraints–the clausemate condition on reflexives, and the necessity of a WhFGD tail in the embedded clause in examples like (3)–and not merely retrieve a c-commanding antecedent, since both *which man* and *Jane* c-command the reflexive in sentences like (3). In our case, if the reflexive is linked to the c-commanding linearly local antecedent, a gender mismatch effect is expected based upon the gender match/mismatch of this NP with the reflexive. On the other hand, if the reflexive is linked to the tail of the WhFGD, due to the parser's respecting the structural constraint on the WhFDG, we should observe a gender mismatch effect due to the *wh*-NP's match/mismatch with the gender of the reflexive.

Dillon et al. (2013) directly compared reflexive antecedent retrieval with a somewhat similar dependency, subject-verb agreement, that also requires feature congruency between words that may be linearly distant from one another. They investigated whether the interference effects found in subject-verb agreement, where an illicit potential antecedent can cause the verb to mistakenly bear incorrect agreement morphology, were also found in reflexive antecedent retrieval, and did not find evidence that they were. Dillon et al. (2013) proposed that, unlike in subject-verb agreement, the antecedents of reflexives are retrieved using solely syntactic cues, with other kinds of cues, such as grammatical gender, being checked against retrieved antecedents only later. On their account, the retrieval system has access to information about which NP in its memory store is the local subject, thus enabling it to be sensitive to c-command as well as locality. On an account of this kind, we would not expect to see an effect of the inaccessible antecedent on reading times of the reflexive.

However, there is a caveat to the preceding discussion. Even if the retrieval system is able to track the identity of the current local subject, it is possible for it to be misled by examples like (3). The grammatically accessible antecedent for the reflexive in (3) is the *wh*-phrase, which is not located inside the immediate local clause containing the reflexive, *to have injured himself*. No local subject is overtly present in this clause at all. The sentences in our experiments thus probe one further level of syntax-sensitivity on the part of the antecedent retrieval system: whether it is able to access the result of the WhFGD resolution process, a posited gap in the subject of the infinitive clause, as a potential reflexive antecedent. There are at least two reasons it might fail to do so.

First, it is possible that the results of WhFGD resolution are simply not represented in a way that is accessible to the reflexive resolution process. This might be the case if the gap/tail of the WhFGD was simply not present in its memory store. Second, it might be the case that the parser is susceptible to what are known as *local coherence effects*, where a parse is adopted that is suitable for only a substring of the input. Note that in examples like (3), if the *wh*-NP is disregarded, the result is the possible sentence *did Jane expect to have injured himself*, in which *Jane* must be the antecedent of the reflexive, contrary to gender congruency. There is evidence that in some contexts the parser can be misled by local coherence effects (e.g., Ferreira et al., 2002; Tabor et al., 2004; Konieczny et al., 2010), and if the result of WhFGD formation is not accessible to the reflexive antecedent retrieval system, it may exhibit such effects in this context as well.

Prior research on reflexive antecedent retrieval in configurations similar to WhFGDs in that they involve an NP associated with a subsequent, unpronounced position similar to a gap has found mixed results. Kwon and Sturt (2015) investigated reflexive antecedent retrieval in the context of nominal control constructions like (14). Control constructions of this kind resemble WhFGDs in that they can involve a dependency between a displaced NP [*Luke* in (14-b)] and a position in an embedded clause [the subject position of *to photograph himself* in (14-b)].

(14)
  a. Luke's order to Sophia to photograph ^*^himself/herself …
  b. Luke's promise to Sophia to photograph himself/^*^herself …

Kwon and Sturt (2015) found an effect of antecedent-reflexive gender mismatch both in nominal control constructions like (14-a), where the accessible antecedent (*Sophia*) was closer to the reflexive than the inaccessible candidate, and in nominal control constructions like (14-b), where the accessible antecedent (*Luke*) was more distant, though the former effect was reliable for more reading-time measures. They interpret this result to indicate that the control relation is processed early on and used to constrain subsequent RD formation.

For our purposes here, the fact that an effect was observed in the control constructions most similar to WhFGDs, those like (14-b) where the antecedent is distant from the embedded clause, suggests that the antecedent retrieval system may be sensitive to agreement mismatch in resolving reflexive-antecedent dependencies even when the antecedent is related to the reflexive via the mediation of a long-distance dependency.

Sturt and Kwon (2015) presented additional results on reflexive antecedent retrieval in nominal control as well as the related construction of *raising*, illustrated in (15).

(15) John seemed to Amy to be kind to himself …

They found evidence of retrieval interference by grammatically inaccessible antecedents for both raising and nominal control, casting further doubt on the possibility that reflexive antecedent search can find an antecedent online whose relation to the reflexive is mediated by a long-distance dependency. Like Sturt (2003)'s early measures, however, they did not find evidence for interference from grammatically inaccessible antecedents in reflexive-antecedent configurations not involving raising or control.

### 1.3. Summary

The resolution of a WhFGD is an active process by which the parser posits the tail of a *wh*-dependency upon encountering grammatically licit positions for it in the input. Similarly, the application of binding conditions in reflexive resolution occurs rapidly in on-line reading. Because of this, sentences containing a *wh*-dependency whose tail constitutes the grammatically-licit antecedent for a reflexive pronoun are an ideal environment for a test for the time course of structure-sensitivity in on-line sentence processing. In particular, these kinds of sentences allow us to test whether backward antecedent search processes are sensitive to fine-grained details of the grammatical representation containing the candidate antecedent NPs. Furthermore, if the timing of effects of accessible and inaccessible antecedents differs, they may may be informative about whether grammatical sensitivity constrains the antecedent search process itself or whether the antecedent search process is itself insensitive to fine-grained syntactic details and syntactic knowledge becomes operative only later as a supplementary cue to filter out impossible antecedent-reflexive relations generated by the antecedent search process. The experiments described below constitute such a test.

## 2. Experiments

### 2.1. Experiment 1

#### 2.1.1. Introduction

Experiment 1 is the principal experiment of this study and serves to test whether reflexive resolution is sensitive to the result of WhFGD resolution. Experiments 2–4, which are reported in subsequent sections below, serve as follow-up experiments to Experiment 1, intended to clarify the interpretation of Experiment 1's results. Like all of the experiments in this study, Experiment 1 tests sentences in which a *wh*-NP occurs at the left edge of a complex sentence involving a matrix clause and an embedded clause, the latter of which contains a reflexive pronoun. In Experiment 1, these sentences look like (16), and all follow the basic template in (17).

(16) [Which cowgirl did Mary/David expect [to have injured herself/himself due to negligence?]]
(17) [Which np_1_ did np_2_ verb [to have verb-ed him-/her-self spillover region]]

By independently varying the reflexive's gender congruency with the linearly local, grammatically inaccessible candidate antecedent NP on the one hand, and with the grammatically accessible (but linearly more distant) *wh*-NP on the other, we use on-line eye-tracking reading measurements of sentences like (16) to investigate whether reflexive antecedent search is immediately sensitive to the presence of a WhFGD or whether it initially considers linearly local but grammatically impossible antecedents.

Much previous work using the gender-mismatch effect as a probe for the parser's establishment of a long-distance dependency has utilized gender-stereotypic nouns like *doctor* and *nurse*. In contrast to this, the experiments reported here all use gender-categorical nouns like *cowgirl* or *uncle* and strongly gendered personal names like *Mary* or *Steven*. The reason for this design decision is that in piloting work, the subject population (Northwestern University undergraduates) was not found to exhibit a measurable gender-mismatch effect in response to gender-stereotypic nouns associated with (stereotypic-)gender mismatched reflexives. We do not speculate here as to the reason for this difference from previously studied populations except to say that it may be connected to changing social attitudes about the appropriateness of different professions for individuals of one or another gender. For our purposes it is sufficient that the study population *does* exhibit a gender-mismatch effect in response to gender-categorical nouns and strongly gendered personal names associated with gender-mismatched reflexives^5^.

#### 2.1.2. Participants

Forty English speaking undergraduates from the Northwestern University community volunteered to participate in this experiment in return for course credit or a small monetary compensation. This experiment, and all experiments reported below, were approved by the Northwestern University Institutional Review Board as compliant with ethical standards for research on human subjects and were run under the protocol *Meaning in Language: Words, Sentences and Inferences (STU00025908)* or *Clausal Ellipsis: Its Structure and Online Processing (STU00082465)*.

#### 2.1.3. Design and materials

Materials consisted of 24 sentences like (18), with a complex *wh*-NP at the left edge associated with the subject position of an embedded non-finite clause, plus 140 filler sentences from unrelated, non-competing experiments. Comprehension questions were asked after 25% of trials in order to motivate the participants to attend to the experiment. This procedure is used in all following experiments as well. The gender match of the reflexive with the *wh*-NP and the linearly closer matrix-clause subject was independently varied in a two-by-two factorial design.

(18) Sample Stimuli
  a. Which cowgirl did Mary expect to have injured herself due to negligence? // *wh*-NP match, local NP match.
  b. Which cowgirl did David expect to have injured herself due to negligence? // *wh*-NP match, local NP mismatch.
  c. Which cowgirl did David expect to have injured himself due to negligence? // *wh*-NP mismatch, local NP match.
  d. Which cowgirl did Mary expect to have injured himself due to negligence? // *wh*-NP mismatch, local NP mismatch.

In this experiment, the embedded clause is non-finite (marked with the infinitival marker *to* and without agreement or independent tense-marking) and the tail of the *wh*-dependency headed by the *wh*-NP terminates in the embedded clause, after the position of the subject of the matrix clause. Although the embedded verbs were not formally normed for transitive or reflexive frame probabilities, they are all judged by the consensus of the native English speaking authors to be obligatorily transitive or highly transitively biased, and none are inherently reflexive. The subject of the matrix clause is thus the closest overt NP to the reflexive, and will consequently be referred to as the *linearly local* candidate antecedent, but because of the long-distance WhFGD between the *wh*-NP and the subject position of the embedded clause (17), only the *wh*-NP can be adopted as the antecedent for the reflexive in the final interpretation of the example^6^. For this reason the *wh*-NP is a *grammatically accessible* antecedent in the terminology we adopt here, and likewise the linearly local NP (the subject of the matrix clause) is a *grammatically inaccessible* antecedent.

In conditions (a) and (c), the reflexive matches the gender of the linearly closer but grammatically inaccessible matrix-clause subject. In conditions (a) and (b), the reflexive matches the gender of the linearly more distant but grammatically accessible *wh*-NP. Full experimental materials for this and all subsequent experiments are available in the online Supplementary Materials.

#### 2.1.4. Predictions

If the process of antecedent search involved in reflexive resolution is sensitive to the output of WhFGD resolution, an early gender-mismatch effect should be observed when the gender of the grammatically accessible *wh*-NP mismatches that of the reflexive [i.e., in conditions (c) and (d)], and no gender-mismatch effect should be observed when the grammatically inaccessible, linearly local NP mismatches the gender of the reflexive, at least in early reading-time measures.

In contrast, if reflexive antecedent search is not sensitive to the output of WhFGD resolution and consists of a retrieval system that is not constrained to consider only grammatical antecedents, early gender-mismatch effects should be observed when the gender of the grammatically inaccessible, linearly local NP mismatches the gender of the reflexive. Depending upon the naive retrieval model adopted, several patterns of effects from the grammatically accessible *wh*-NP might be observed. Effects of gender mismatch with the *wh*-NP may be predicted to be observed only in later measures, if subjects select the linearly closest candidate antecedent on their initial parse. This might be the case if subjects are initially misled into a locally-coherent but globally ungrammatical parse (Ferreira et al., 2002; Tabor et al., 2004; Konieczny et al., 2010) in which the *wh*-phrase is not assigned an interpretation, as discussed above. In this case effects of the *wh*-NP would be expected to follow those of the linearly local candidate antecedent. On the other hand, if the reflexive antecedent retrieval system is a cue-based system that is not constrained to consider only grammatical antecedents, an effect of gender mismatch with the linearly local but grammatically inaccessible candidate antecedent should interact with that of the grammatically accessible *wh*-NP. In particular, the slowdown effect due to gender mismatch with the *wh*-NP should be ameliorated in the presence of a gender-matching inaccessible antecedent, because a cue-based retrieval system that is not restricted to consider only syntactically accessible candidate antecedents should be able to retrieve the gender-matching but grammatically inaccessible candidate antecedent.

Importantly however, a linguistically-naive antecedent retrieval process, whether cue-based or otherwise, should always show effects of the grammatically inaccessible, linearly local NP if any gender-mismatch effects are measurable at all. This is because such a process can by definition not distinguish potential candidate antecedents based upon the syntactic configurations in which they occur. For this reason, an effect of the grammatically accessible *wh*-NP in this experiment in the absence of an effect of the grammatically inaccessible, linearly local NP should be a clear signal of structure-sensitivity in the reflexive antecedent search mechanism.

#### 2.1.5. Data analysis

Using a tower-mounted EyeLink1000 eye-tracker, gaze was recorded and manually corrected for vertical drift. Fixations shorter than 80 ms were incorporated into adjacent fixations, and fixations longer than 2000 ms were excluded from analysis. The following analysis is based on four eye-tracking measures: first fixation duration, first pass duration, regression path duration, and re-read time. First fixation measures are based on the duration of the first time a fixation occurs within the region. First pass times include all time spent within the region before the first instance of the gaze exiting the region, either to the left or the right. Regression path duration is calculated by summing the times spent within the region and all time after exiting the region to the left until the first instance that the gaze exits to the right of the region. Re-read time is the sum of time spent within the region after the first time the gaze exits the region.

For the purposes of this study, we will concentrate on two regions of interest: the critical region containing the reflexive anaphor [e.g., *herself* in (19)], and the spillover region containing the remaining words on that line before the carriage return [e.g., *for unimportant* in (19)]. The stimuli were all displayed on two lines, due to character length limitations of the presentation software. The carriage returns were all in the same location and included in the post-spillover region, which is not analyzed in this study due to the complexity of interpreting fixations in regions that contain line breaks.

(19) Which saleswoman did Margaret presume to have excused herself for unimportant reasons?

In line with discussion in Barr et al. (2013), analyses were conducted by comparing a converging maximally inclusive linear mixed effects regression (LMER) model to a reduced model, i.e., a model with the same structure as the maximal model but with a single effect of interest removed from the fixed effects structure. Intercepts (β) and standard error (S.E.) were calculated from the maximal model. Maximal and reduced models were then compared by ANOVA to calculate the χ^2^ and significance (α = 0.05), reported in **Table 2**. The ideal maximal model for the critical region consisted of two independent factors (gender congruency with the *wh*-phrase; gender congruency with the local NP), and one additional fixed factor (presentation order). Intercepts were allowed to vary across subjects and items. We also allowed for the slopes of the following effects to vary across subjects and items: gender congruency of the *wh*-phrase, gender congruency of the local antecedent, the interaction of these two factors, and the presentation order. In cases where the maximal model failed to converge, the random effects correlation parameters were removed from the random effects structure (thus necessitating removal from reduced models as well). All models converged with either the ideal maximal model^7^, or with the random effects correlations removed, as suggested in Bates et al. (2015). Data were contrast coded with conditions summing to 0 (i.e., *wh*- and local congruency conditions were coded as 0.5 or −0.5, respectively.) This coding scheme and analytical method is used for all experiments in this study. Table 1 contains the means and standard errors in milliseconds of reading times. These measures were calculated after manual vertical alignment of fixations. For statistical analysis, converging maximal linear mixed effect models were compared via ANOVA to depleted models of the same structure, but with a term of interest removed. χ^2^-values and their corresponding *p*-values are reported in Table 2, alongside the estimates and standard errors calculated from the corresponding maximal model. Bold values indicate that the comparison reached significance.

**Table 1:** **Means (and Standard Errors) for Experiment 1**.

**Region**		**Critical region**	**Spillover region**
**Wh-phrase**	**Local NP**		
**FIRST FIXATION**			
Match	Match	207 (6)	199 (5)
Match	Mismatch	203 (5)	214 (6)
Mismatch	Match	212 (5)	209 (7)
Mismatch	Mismatch	221 (6)	212 (7)
**FIRST PASS**			
Match	Match	221 (7)	312 (14)
Match	Mismatch	225 (7)	338 (15)
Mismatch	Match	238 (8)	335 (16)
Mismatch	Mismatch	247 (9)	343 (15)
**REGRESSION PATH**			
Match	Match	314 (23)	666 (64)
Match	Mismatch	293 (17)	680 (63)
Mismatch	Match	331 (25)	757 (57)
Mismatch	Mismatch	404 (34)	931 (78)
**RE-READ TIME**			
Match	Match	395 (28)	434 (42)
Match	Mismatch	297 (23)	391 (28)
Mismatch	Match	376 (28)	463 (37)
Mismatch	Mismatch	397 (25)	457 (31)

**Table 2:** **Combined ANOVA and LME results for Experiment 1**.

**Region**	**Effect**	**Estimate**	**Std error**	**χ^2^ (df)**	***p*-value**
**FIRST FIXATION**					
*Critical*	*wh*−NP	−12.71	6.30	3.71 (1)	0.054
	local NP	−4.15	6.21	0.44 (1)	>0.1
	interaction	16.09	12.11	1.69 (1)	>0.1
*Spillover*	*wh*−NP	−4.23	7.03	0.36 (1)	>0.1
	local NP	−8.17	6.01	1.80 (1)	>0.1
	interaction	−10.05	13.80	0.52 (1)	>0.1
**FIRST PASS**					
*Critical*	*wh*−NP	−**19.55**	**8.32**	**6.65** (1)	**0.010**
	local NP	−6.15	8.84	1.36 (1)	>0.1
	interaction	9.58	16.83	0.36 (1)	>0.1
*Spillover*	*wh*-NP	−10.00	14.34	0.47 (1)	>0.1
	local NP	−21.83	13.83	2.32 (1)	>0.1
	interaction	−19.29	25.59	0.56 (1)	>0.1
**REGRESSION PATH**					
*Critical*	*wh*−NP	−59.88	30.65	3.43 (1)	0.064
	local NP	−24.55	24.95	0.94 (1)	>0.1
	interaction	90.75	49.05	3.40 (1)	0.065
*Spillover*	*wh*-NP	−**159.70**	**72.40**	**4.33** (1)	**0.037**
	local NP	−102.67	78.77	1.62 (1)	>0.1
	interaction	121.97	115.73	1.09 (1)	>0.1
**RE-READ TIME**					
*Critical*	*wh*-NP	−39.57	27.45	2.00 (1)	>0.1
	local NP	24.80	27.82	0.78 (1)	>0.1
	interaction	91.31	51.72	2.87 (1)	0.090
*Spillover*	*wh*-NP	−29.80	34.60	0.71 (1)	>0.1
	local NP	26.54	34.26	0.59 (1)	>0.1
	interaction	27.19	68.70	0.16 (1)	>0.1

#### 2.1.6. Results

In the critical region, i.e., at the reflexive pronoun, we found a significant main effect of gender congruency between the *wh*-phrase and the reflexive, with matched gender read faster than mismatched gender, for first pass reading time [β = −19.55, S.E. = 8.32, χ^2^(1) = 6.65, *p* = 0.010]. This suggests that the parser is trying to form a dependency between the *wh*-phrase and the reflexive pronoun. When it successfully forms the dependency in the *wh*-phrase gender match condition, the reading time at the critical region is faster than when it is unsuccessful in the *wh*-phrase gender mismatch condition.

**Figure 1:**
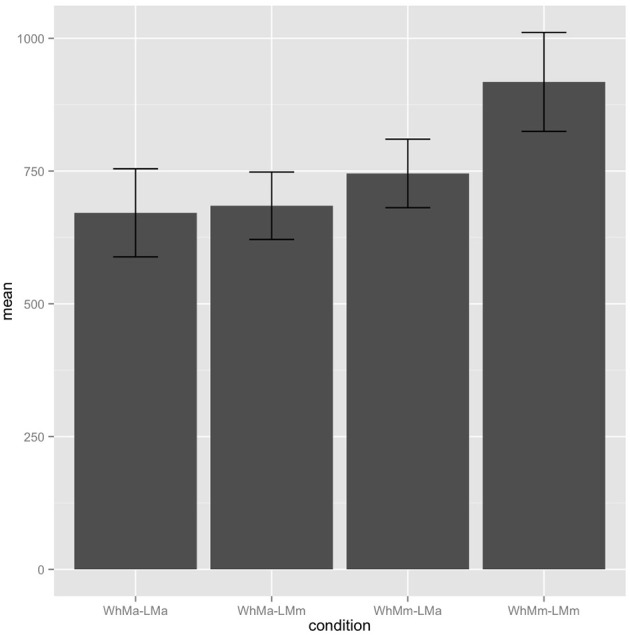
**Experiment 1 spillover region regression path durations**.

In the spillover region, we observe a significant main effect of gender congruency between the *wh*-phrase and the reflexive, with matched gender read faster than mismatched gender for regression path duration [β = −159.70, S.E. = 72.40, χ^2^(1) = 4.33, *p* = 0.037], (Figure 1). No other effects reached significance.

There were, however, marginal interactions of *wh*-phrase gender congruence with local NP congruence in the regression path duration and re-reading time in the critical region, such that the mismatch-mismatch condition was read more slowly. Although this interaction was not statistically significant it is consistent with the predictions of some unconstrained cue-based models of antecedent retrieval. On an explanation of this kind, the parser would attempt to associate the reflexive with all possible candidate antecedents in parallel and experience extra difficulty when no gender-congruent antecedent is found in its memory store. In the absence of a significant effect, this is of course a purely speculative suggestion.

The main effect we observe in the spillover region is consistent with the effect at the critical region and supports the hypothesis that the parser represents the tail of the *wh*-dependency and is thus able to connect the *wh*-phrase and the reflexive pronoun. This suggests that the presence of the WhFGD is accessible to the process of RD resolution. In other words, since the parser has already linked the *wh*-phrase with the gap, the search for the RD does not allow the parser to consider the interpretation in which the linearly closest antecedent (i.e., the proper name) is linked with the gap. The effect of gender mismatch of the *wh*-phrase in the absence of an effect of the linearly local but grammatically inaccessible candidate antecedent supports the hypothesis that the reflexive antecedent retrieval system is constrained to consider only grammatically accessible antecedents. However, the marginal interaction with the gender-match/mismatch of the linearly local candidate antecedent suggests a possible signature of a cue-based retrieval system that is not constrained to consider only grammatically accessible antecedents, which forms much of the motivation for Experiment 2.

In addition to the reflexive interpretation proper, the English pronouns ending in *-self* have at least two other interpretations which are subject to different syntactic constraints^8^. In an *emphatic* reading of a *-self* -type pronoun in English, the pronoun, though formally reflexive, does not have a properly reflexive reading (roughly, indicating that the object of the verb refers to the same entity as the subject). Emphatic reflexives instead have a focus-related meaning emphasizing that some entity referred to by an NP associated with the reflexive was involved in the event described by the sentence, rather than any other entity that might have been involved in the event. So in (20-a), the emphatic reflexive *himself* is associated with the matrix subject *John* and serves to emphasize that John's expectation was that he himself, and not someone else, would have injured the cowgirl.

(20)
  a. John expected to have injured the cowgirl himself.
  b.^*^John expected had injured the cowgirl himself.

In an *anti-assistive* reading of a *-self* -type pronoun, the *-self* -type pronoun serves to indicate that the agent of the sentence performed the action in question *without help*, so in (20-a), such a reading would mean that John expected to have received no assistance in injuring the cowgirl. Because control into finite embedded clauses is impossible in English (20-b), emphatic and anti-assistive reflexive readings for sentences like the stimuli for Experiment 2 (discussed below), with finite embedded clauses, are not possible.

### 2.2. Experiment 2

#### 2.2.1. Introduction

In order to demonstrate that the effect of the *wh*-NP observed in Experiment 1 is, in fact, a consequence of the *wh*-dependency and not some other factor, we should replicate these results in a syntactically different context, but one that is similar in all respects that this account predicts to be relevant for the pattern of results observed in Experiment 1: namely, the presence of a dependency tail associated with the sentence-initial *wh*-NP after the linearly closest candidate antecedent. This is the primary purpose of Experiment 2.

Experiment 2 also serves to distinguish the possibility that the marginal interactions with the gender congruence of the local NP result from retrieval difficulty from the possibility that they result from the parser's later consideration of the dispreferred non-reflexive readings for the *-self* pronoun.

#### 2.2.2. Participants

Forty English speaking undergraduates from the Northwestern University community volunteered to participate in this experiment in return for course credit or a small monetary compensation.

#### 2.2.3. Design and materials

Materials for Experiment 2 consisted of 24 target sentences, plus 90 filler sentences from unrelated experiments. The target stimuli used in Experiment 2 are based upon those used in Experiment 1, with one relevant difference. While the target stimuli from Experiment 1 include non-finite embedded clauses, those in Experiment 2 use finite embedded clauses, as exemplified in (21).

(21) Sample Stimuli
  a. Which cowgirl did Mary expect had injured herself due to negligence? // *wh*-NP match, local NP match.
  b. Which cowgirl did David expect had injured herself due to negligence? // *wh*-NP match, local NP mismatch.
  c. Which cowgirl did David expect had injured himself due to negligence? // *wh*-NP mismatch, local NP match.
  d. Which cowgirl did Mary expect had injured himself due to negligence? // *wh*-NP mismatch, local NP mismatch.

This difference has two related effects on the possible behavior of the parser in these sentences. First, because finite complement clauses in English do not permit control readings (22), there is no potential locally coherent substring of these examples in which the grammatically inaccessible, linearly local candidate antecedent NP is a grammatical antecedent for the reflexive. Given that effects of the linearly local candidate antecedent were not observed in Experiment 1, this difference is not expected to influence reading time measures.

(22) ^*^Susan expected had injured herself.

A related but more important difference is that, precisely *because* a control reading is not possible for examples like those in (21), these examples do not admit of intensifier readings for the reflexive. For this reason, then, there is no grammatical possibility of linking the reflexives in the embedded clause with the matrix subject.

It is not clear how the possibility of an intensifier reflexive reading might have contaminated the primary results of Experiment 1, given that the observed effects were not compatible with such a reading (i.e., they did not indicate that participants were attempting to associate the reflexive with the matrix subject rather than with the *wh*-NP). However, because intensifier reflexives are subject to somewhat different syntactic constraints than reflexives proper, it was deemed worthwhile to ensure that a similar pattern of results obtained in the absence of any possibility of such a reading. Moreover, if the marginal interactions reported above do result from the parser's consideration of an intensifier reading for the reflexive, they should disappear in a context where this is not possible. In contrast, if they arise from interference in the antecedent retrieval process proper, they should be expected to persist.

#### 2.2.4. Predictions

The results of Experiment 2 are predicted to be broadly similar to those of Experiment 1: namely, if reflexive resolution is sensitive to presence of a WhFGD and constrained to consider only grammatically accessible antecedents, a gender-mismatch effect should be observed when the gender of the grammatically accessible *wh*-NP mismatches that of the reflexive [i.e., in conditions (c) and (d)], and no gender-mismatch effect should be observed when the grammatically inaccessible, linearly local NP mismatches the gender of the reflexive. If reflexive antecedent search is not constrained to consider only grammatically accessible antecedents, gender-mismatch effects should be observed when the gender of the grammatically inaccessible, linearly local NP mismatches the gender of the reflexive. If this is because of the antecedent search process's susceptibility to linear closeness, gender mismatch effects from the linearly local candidate antecedent should precede any from the *wh*-NP. If instead antecedent retrieval consists of a cue-based retrieval system that is able to consider ungrammatical reflexive antecedents, gender mismatch effects of both candidate antecedents should interact in such a way that the slowdown effect induced by mismatch with the *wh*-NP is ameliorated in the presence of a gender-matching ungrammatical candidate antecedent.

However, because in the finite embedded clauses used in Experiment 2 no control reading is possible, it is not possible to interpret the reflexive in the examples used in Experiment 2 as an intensifier reflexive linked to the matrix subject, so this experiment may constitute a cleaner test of the role of the binding constraints in reflexive antecedent search. It is not expected that the pattern of effects in this experiment will differ from that in Experiment 1; if it does, this would cast doubt upon an explanation of the effect of the *wh*-NP in Experiment 1 in terms of the parser's online sensitivity to find-grained syntactic constraints.

#### 2.2.5. Data analysis

The analysis of the data gathered in this experiment was carried out in much the same way as in Experiment 1. The critical region corresponds to the reflexive pronoun (*herself*) and the spillover region corresponds to *for unimportant* in the example below. Since the stimuli used in this experiment are adapted from Experiment 1, the same limitations on region size due to line breaks constrained the spillover region.

(23) Which saleswoman did Margaret presume had excused herself for unimportant reasons?

Table 3 contains the means and standard errors in milliseconds of reading times. These measures were calculated after manual vertical alignment of fixations. For statistical analysis, converging maximal linear mixed effect models were compared via ANOVA to depleted models of the same structure, but with a term of interest removed. χ^2^-values and their corresponding *p*-values are reported in Table 4, alongside the estimates and standard errors calculated from the corresponding maximal model. The ideal maximal structure contains the same terms as in Experiment 1, and in cases where a maximal or depleted model did not converge, additional terms were removed in the order specified above.

**Table 3:** **Means (and Standard Errors) for Experiment 2**.

**Region**		**Critical region**	**Spillover region**
**Wh-phrase**	**Local NP**		
**FIRST FIXATION**			
Match	Match	209 (6)	204 (5)
Match	Mismatch	214 (5)	208 (6)
Mismatch	Match	222 (6)	219 (6)
Mismatch	Mismatch	213 (4)	213 (6)
**FIRST PASS**			
Match	Match	223 (7)	293 (12)
Match	Mismatch	238 (8)	319 (14)
Mismatch	Match	250 (9)	356 (18)
Mismatch	Mismatch	237 (8)	348 (18)
**REGRESSION PATH**			
Match	Match	461 (55)	1860 (119)
Match	Mismatch	454 (45)	1867 (112)
Mismatch	Match	620 (66)	2656 (171)
Mismatch	Mismatch	611 (73)	2744 (197)
**RE-READ TIME**			
Match	Match	300 (32)	382 (38)
Match	Mismatch	335 (26)	381 (30)
Mismatch	Match	463 (33)	477 (35)
Mismatch	Mismatch	426 (28)	494 (34)

**Table 4:** **Combined ANOVA and LME results for Experiment 2**.

**Region**	**Effect**	**Estimate**	**Std error**	**χ^2^ (df)**	***p*-value**
**FIRST FIXATION**					
*Critical*	*wh*-NP	−6.30	5.05	1.55 (1)	>0.1
	local NP	3.19	5.48	0.34 (1)	>0.1
	interaction	−11.09	10.79	1.02 (1)	>0.1
*Spillover*	*wh*-NP	−8.79	7.92	1.20 (1)	>0.1
	local NP	3.31	6.05	0.30 (1)	>0.1
	interaction	−13.48	12.70	1.11 (1)	>0.1
**FIRST PASS**					
*Critical*	*wh*-NP	−13.11	7.44	3.08 (1)	0.079
	local NP	−0.05	8.30	0.0001 (1)	>0.1
	interaction	0.22	7.75	2.60 (1)	>0.1
*Spillover*	*wh*-NP	−**42.53**	**13.92**	**8.21** (1)	**0.004**
	local NP	−11.07	13.60	0.66 (1)	>0.1
	interaction	−33.21	31.13	1.11 (1)	>0.1
**REGRESSION PATH**					
*Critical*	*wh*-NP	−140.07	81.96	2.80 (1)	0.094
	local NP	−13.73	60.67	0.05 (1)	>0.1
	interaction	41.56	132.41	0.10 (1)	>0.1
*Spillover*	*wh*-NP	−**831.51**	**140.41**	**21.11** (1)	**< 0.001**
	local NP	−91.67	135.46	0.45 (1)	>0.1
	interaction	152.58	309.65	0.24 (1)	>0.1
**RE-READ TIME**					
*Critical*	*wh*-NP	−**129.22**	**31.34**	**12.43** (1)	**< 0.001**
	local NP	−7.94	30.75	0.06 (1)	>0.1
	interaction	−80.55	62.04	1.61 (1)	>0.1
*Spillover*	*wh*-NP	−**107.00**	**43.43**	**5.13 (1)**	**0.023**
	local NP	−14.26	40.13	0.12 (1)	>0.1
	interaction	−6.61	82.07	0.006 (1)	>0.1

#### 2.2.6. Results

In the critical region, the only observed effects are in re-read time. We observe a significant main effect of the *wh*-phrase, with the gender matched condition read faster than gender mismatched conditions [β = −129.22, S.E. = 31.34, χ^2^(1) = 12.43, *p* < 0.001]. This is consistent with the observations in Experiment 1, that the local NP is not considered as a candidate antecedent for the reflexive pronoun, despite its linear proximity. No other effects reached significance.

**Figure 2:**
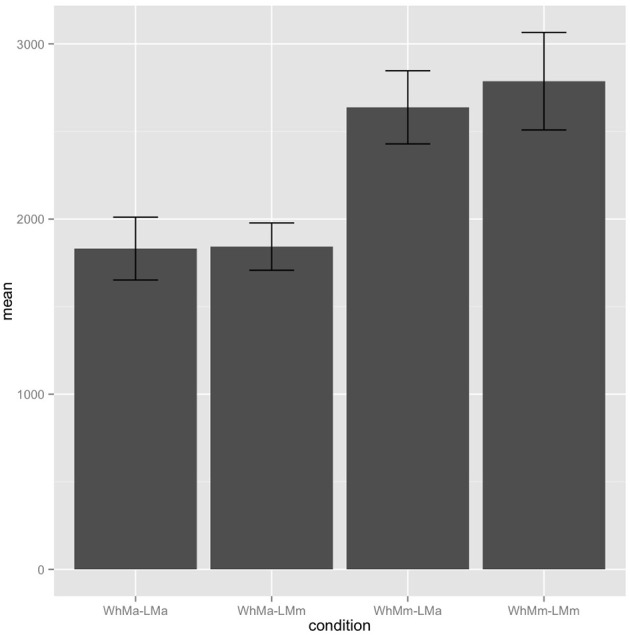
**Experiment 2 spillover region regression path durations**.

The spillover region displays a similar pattern of effects, with the addition of significant main effects of *wh*-phrase observed in first pass reading time, regression path duration (Figure 2), and re-read time, with gender match between the *wh*-phrase and the reflexive pronoun read faster than gender mismatch [first pass: β = −42.53, S.E. = 13.92, χ^2^(1) = 8.21, *p* = 0.004; regression path: β = −831.51, S.E. = 140.41, χ^2^(1) = 21.11, *p* < 0.001], as in the case for re-read time [β = −107.00, S.E. = 43.43, χ^2^(1) = 5.13, *p* = 0.023].

As before, this indicates that the gender of the *wh*-phrase is somehow represented at the tail of the WhFGD, which is then being accessed during the reflexive antecedent search. These results are compatible with our observations in Experiment 1. As such, we can confirm that the gender mismatch effects observed in the critical region and spillover region in both Experiments 1 and 2 are due to the ability of the parser to form a dependency between the reflexive and the gap, although for different reasons. There were no marginal effects of the linearly local candidate antecedent NP in this experiment, unlike in Experiment 1, suggesting that the intensifier reading explanation for those effects in Experiment 1 may be on the right track, rather than an interpretation in terms of failed cue-based retrieval. This is of course merely speculation, given that the effects in question do not reach statistical significance.

### 2.3. Experiment 3

#### 2.3.1. Introduction

Experiment 3 (as well as Experiment 4, discussed below) serves as a check to ensure that the difference observed in Experiments 1 and 2 between the effect of gender match/mismatch of the reflexive and the *wh*-NP and of the reflexive and the linearly closer NP is not due to some difference between the way *wh*-NPs and personal names are processed in general. For example, the results of Martin and McElree (2011) indicate that *wh*-NPs may have a higher prominence in memory, inasmuch as they are candidates for antecedent retrieval, than other categories. Therefore, there is a possibility that the results of Experiments 1 and 2 are not demonstrating grammar-sensitivity on the part of the parser's reflexive antecedent search process, but are instead merely demonstrating that *wh*-NPs are treated differently in memory than other NPs in some way that causes them to induce gender mismatch effects on subsequently encountered reflexives.

For this reason, in Experiment 3, the WhFGD originating in the sentence-initial *wh*-NP does not span across the linearly local NP but terminates before it, in the matrix clause, as in (24).

(24) Which cowgirl expected Mary to have injured herself due to negligence?

This has the effect that the *wh*-NP, though equally distant from the reflexive, is not its grammatical antecedent. If the effect of the *wh*-NP observed in Experiments 1 and 2 is due to a general high salience of *wh*-NPs in memory, the pattern of effects in this experiment should be largely the same here. On the other hand, if the effect of the *wh*-NP on RTs at and following the reflexive in Experiments 1 and 2 is due to the parser's sensitivity to the presence of a WhFGD intervening between the more linearly local candidate antecedent and the reflexive, the linearly local candidate antecedent should modulate RTs at the reflexive in this experiment rather than the *wh*-NP.

#### 2.3.2. Participants

Forty English speaking undergraduates from the Northwestern University community volunteered to participate in this experiment in return for course credit or a small monetary compensation.

#### 2.3.3. Design and materials

Materials for Experiment 3 consist of 24 target sentences and 88 filler sentences from unrelated experiments. Experiment 3 (as well as Experiment 4, discussed below) serves as a check to ensure that the difference observed in Experiments 1 and 2 between the effect of gender match/mismatch of the reflexive and the *wh*-NP and of the reflexive and the linearly closer NP is not due to some difference between the way *wh*-NPs and personal names are processed in general. That is, there is a possibility that the results of Experiments 1 and 2 are not demonstrating grammar-sensitivity on the part of the parser's reflexive antecedent search process, but are instead merely demonstrating that *wh*-NPs are treated differently in memory than other NPs in some way that causes them to induce gender mismatch effects on subsequently encountered reflexives. For this reason, in Experiment 3, the WhFGD originating in the sentence-initial *wh*-NP does not span across the linearly local NP but terminates before it, as in (25).

(25) Sample Stimuli
  a. Which cowgirl expected Mary to have injured herself due to negligence? // *wh*-NP match, local NP match.
  b. Which cowgirl expected David to have injured herself due to negligence? // *wh*-NP match, local NP mismatch.
  c. Which cowgirl expected David to have injured himself due to negligence? // *wh*-NP mismatch, local NP match.
  d. Which cowgirl expected Mary to have injured himself due to negligence? // *wh*-NP mismatch, local NP mismatch.

If the effect of the gender match of the *wh*-NP in Experiments 1 and 2 is to be attributed to the parser's sensitivity to the WhFGD between the *wh*-NP and the embedded clause, this effect should go away when the WhFGD is not associated with the embedded clause but instead with the matrix clause, as in (25). On the other hand, if the role of the *wh*-NP in modulating reading times of the reflexive is due to a high overall salience of *wh*-NPs in memory, it should persist in this experiment.

#### 2.3.4. Predictions

If the patterns of effects observed in Experiments 1 and 2–broadly, effects of the *wh*-NP's gender match/mismatch with the reflexive on the reading times of the reflexive–is due to the parser's grammatical sensitivity to the presence of a long-distance WhFGD between the sentence-initial *wh*-word and the embedded clause, the result in this experiment should be very different. In particular, because no such long-distance WhFGD between the sentence-initial *wh*-word and the embedded clause is present in the stimuli used in Experiment 3, no effect of the *wh*-NP's gender match/mismatch with the reflexive should be observed in this experiment. On the other hand, if the effect of the *wh*-NP on the reading time of the reflexive in Experiments 1 and 2 is due, in whole or in part, to a difference between the way that the parser treats previously-processed *wh*-NPs and the way it treats previously-processed personal names, an effect of the gender match/mismatch of the *wh*-NP should be observed in this experiment as well. If the results of Experiments 1 and 2 are due entirely to a difference between the behavior of previously-processed *wh*-NPs and personal names, then, the results of this experiment should be the same as those of Experiments 1 and 2. If a difference between the behavior of previously-processed *wh*-NPs and personal names is a contributor to the pattern of results in Experiments 1 and 2 but not the sole driver of the effect, with grammar-sensitivity of the parser also being implicated, then an effect of the gender match/mismatch of both candidate antecedent NPs, the *wh*-NP and the linearly local NP, should be observed. As above, if the antecedent retrieval system is a cue-based retrieval system that is not constrained to consider only grammatical antecedents, an interaction effect should be observed such that the gender mismatch effect due to the grammatically accessible antecedent (in this case, the linearly local antecedent rather than the *wh*-NP) should be ameliorated provided the other candidate antecedent is gender-matched with the reflexive.

#### 2.3.5. Data analysis

The analysis of the data gathered in this experiment was carried out in much the same way as in the previous two experiments. The critical region corresponds to the reflexive pronoun (*herself*) and the spillover region corresponds to *for unimportant* below. The stimuli used in this experiment are again adapted from Experiment 1 and the same limitations on region size due to line breaks constrained the spillover region. The critical difference between the stimuli in Experiments 1 and 2 and the current set is that the *wh*-phrase is no longer accessible to the reflexive pronoun. Rather, the local antecedent is the globally coherent and accessible antecedent.

(26) Which saleswoman presumed Margaret to have excused herself for unimportant reasons?

Table 5 displays the means and standard errors in milliseconds of reading times, calculated after manual vertical alignment of fixations. For statistical analysis, converging maximal linear mixed effect models were compared via ANOVA to depleted models of the same structure, but with a term of interest removed. χ^2^-values and their corresponding *p*-values are reported in Table 6, alongside the estimates, and standard errors calculated from the corresponding maximal model. The ideal maximal structure contains the same terms as in previous experiments, and in cases where a maximal or depleted model did not converge, additional terms were removed in the order previously specified.

**Table 5:** **Means (and Standard Errors) for Experiment 3**.

**Region**		**Critical region**	**Spillover region**
**Wh-phrase**	**Local candidate**		
**FIRST FIXATION**			
Match	Match	191 (4)	195 (5)
Match	Mismatch	200 (5)	212 (6)
Mismatch	Match	198 (4)	200 (5)
Mismatch	Mismatch	200 (4)	207 (5)
**FIRST PASS**			
Match	Match	205 (5)	319 (13)
Match	Mismatch	220 (7)	332 (13)
Mismatch	Match	217 (6)	307 (11)
Mismatch	Mismatch	211 (5)	345 (16)
**REGRESSION PATH**			
Match	Match	397 (41)	2068 (109)
Match	Mismatch	527 (59)	2907 (146)
Mismatch	Match	412 (52)	2117 (116)
Mismatch	Mismatch	423 (42)	2968 (181)
**RE-READ TIME**			
Match	Match	305 (21)	333 (25)
Match	Mismatch	413 (26)	482 (31)
Mismatch	Match	339 (35)	394 (31)
Mismatch	Mismatch	447 (33)	568 (69)

**Table 6:** **Combined ANOVA and LME results for Experiment 3**.

**Region**	**Effect**	**Estimate**	**Std error**	**χ^2^ (df)**	***p*-value**
**FIRST FIXATION**					
*Critical*	*wh*-NP	−4.64	4.76	0.93 (1)	>0.1
	local	−7.79	4.25	3.24 (1)	0.072
	interaction	−8.00	10.64	0.55 (1)	>0.1
*Spillover*	*wh*-NP	−0.35	6.10	0.003 (1)	>0.1
	local	−10.98	6.27	2.76 (1)	0.097
	interaction	−8.22	10.47	0.60 (1)	>0.1
**FIRST PASS**					
*Critical*	*wh*-NP	−0.57	6.29	0.01 (1)	>0.1
	local	−5.28	7.01	0.55 (1)	>0.1
	interaction	−24.08	16.89	1.95 (1)	>0.1
*Spillover*	*wh*-NP	2.24	13.87	0.026 (1)	>0.1
	local	−25.92	13.24	3.44 (1)	0.064
	interaction	23.27	26.80	0.73 (1)	>0.1
**REGRESSION PATH**					
*Critical*	*wh*-NP	43.67	44.18	0.98 (1)	>0.1
	local	−74.27	45.67	2.58 (1)	>0.1
	interaction	−111.73	96.88	1.29 (1)	>0.1
*Spillover*	*wh*-NP	−29.80	120.90	0.06 (1)	>0.1
	local	−**844.80**	**117.30**	**27.58** (1)	**< 0.001**
	interaction	113.20	253.30	0.20 (1)	>0.1
**RE-READ TIME**					
*Critical*	*wh*-NP	−44.40	29.08	2.18 (1)	>0.1
	local	−**100.51**	**35.59**	**6.62** (1)	**0.010**
	interaction	3.84	81.52	0.002 (1)	>0.1
*Spillover*	*wh*-NP	−79.11	59.80	1.64 (1)	>0.1
	local	−**164.91**	**69.38**	**4.97** (1)	**0.026**
	interaction	27.40	141.89	0.04 (1)	>0.1

#### 2.3.6. Results

**Figure 3:**
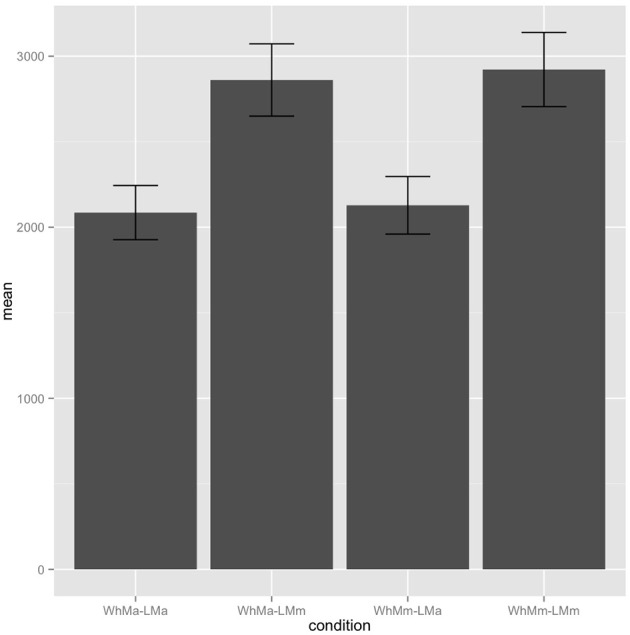
**Experiment 3 spillover region regression path durations**.

In this experiment, we observe the expected reverse in effect source, now with the gender of the local NP influencing reading times in the critical region. Here, we observe a main effect of local NP in the critical region's re-read time, with the gender matched conditions read faster than gender mismatched condition [β = −100.51, S.E. = 35.59, χ^2^(1) = 6.62, *p* = 0.010]. We also observe a main effect of gender mismatch in the regression path duration (Figure 3) and re-read time) in the spillover region [regression path: β = −844.80, S.E. = 117.30, χ^2^(1) = 27.58, *p* < 0.001; re-read time: β = −164.91, S.E. = 69.38, χ^2^(1) = 4.97, *p* = 0.026]. No other effects reached significance. Note however that all marginal effects are of the local NP, consistent with the parser only considering this NP as a potential reflexive antecedent. Thus, this supports the hypothesis that the results of Experiments 1 and 2 are due to the RD resolution process being sensitive to the presence of the WhFGD, rather than being due to some general property of *wh*-NPs as candidate antecedents.

### 2.4. Experiment 4

#### 2.4.1. Introduction

Experiment 4 serves primarily to complete the paradigm explored in Experiments 1–3, so that over the course of all four experiments all combinations of *finite vs. nonfinite embedded clause* and *matrix interpretation of wh-word vs. embedded WhFGD tail* are investigated. The results of this experiment are not expected to differ from those of Experiment 3 except that, because of certain differences between finite and non-finite embedded clauses, as discussed below, the effect of the local candidate antecedent may be stronger in Experiment 4 than in Experiment 3.

#### 2.4.2. Participants

Twenty English speaking undergraduates from the Northwestern University community volunteered to participate in this experiment in return for course credit or a small monetary compensation^9^.

#### 2.4.3. Design and materials

The design of Experiment 4 is substantially the same as that of Experiment 3. The materials consist of 24 target sentences, plus 144 filler sentences from unrelated experiments. Like in Experiment 3, the sentence-initial *wh*-word is associated not with the embedded clause but with the matrix clause, and consequently it is not associated with a dependency tail intervening between the linearly local candidate antecedent and the reflexive. The difference between Experiments 3 and 4 is that in Experiment 4, as in Experiment 2, the embedded clause is finite rather than non-finite, as in (27).

(27) Sample Stimuli
  a. Which cowgirl expected Mary had injured herself due to negligence? // *wh*-NP match, local NP match.
  b. Which cowgirl expected David had injured herself due to negligence? // *wh*-NP match, local NP mismatch.
  c. Which cowgirl expected David had injured himself due to negligence? // *wh*-NP mismatch, local NP match.
  d. Which cowgirl expected Mary had injured himself due to negligence? // *wh*-NP mismatch, local NP mismatch.

#### 2.4.4. Predictions

Because the only difference between Experiments 4 and 3 is the finiteness of the embedded clause, the result of this experiment is not expected to differ from that of Experiment 3. In particular, in this experiment as well, no long-distance WhFGD between the sentence-initial *wh*-word and the embedded clause is present in the stimuli used. Therefore, no effect of the *wh*-NP's gender match/mismatch with the reflexive should be observed in this experiment if the effect of the *wh*-NP's gender match/mismatch with the reflexive observed in the results of Experiments 1 and 2 is due to the parser's grammatical sensitivity to the presence of a long-distance WhFGD whose tail intervenes between the linearly closer NP and the reflexive. Likewise, if the effect of the *wh*-NP's gender match/mismatch with the reflexive is due to a general processing difference between *wh*-NPs and other NPs, it should be observed in this experiment as well. As in the preceding experiments, if the antecedent retrieval system is a cue-based retrieval system that is not constrained to consider only grammatical antecedents, an interaction effect should be observed such that the gender mismatch effect due to the grammatically accessible antecedent should be ameliorated provided the other candidate antecedent is gender-matched with the reflexive.

However, one possible small difference may be observed because of the similarity of embedded finite clauses to matrix clauses in English. Note that in example (27), if the initial words *which cowgirl expected* were omitted, the example would be the entirely grammatical matrix declarative sentences *Mary/David had injured himself/herself due to negligence*, until the presence of the question mark. It is conceivable that in these examples, for this reason, the association of the reflexive with the linearly local NP may be easier for the parser to detect, because of the similarity of these examples to simple matrix sentences in which there is only one candidate antecedent. If something like this is the case, we might expect the effect of the local candidate NP to reach significance for more reading-time measures than in Experiment 3.

#### 2.4.5. Data analysis

The analysis of the data gathered in this experiment was carried out in much the same way as in the previous three experiments. The critical region corresponds to the reflexive pronoun (*herself*) and the spillover region corresponds to *for unimportant*. Using the same design as in Experiment 3, the *wh*-phrase (i.e., *Which saleswoman*) is inaccessible to the reflexive pronoun as an antecedent, while the local antecedent (i.e., *Margaret*) is accessible. The limitations on region size due to line breaks constrained the spillover region, as in the previous experiments.

(28) Which saleswoman presumed Margaret had excused herself for unimportant reasons?

Table 7 displays the means and standard errors in milliseconds of reading times, calculated after manual vertical alignment of fixations. For statistical analysis, converging maximal linear mixed effect models were compared via ANOVA to depleted models of the same structure, but with a term of interest removed. χ^2^-values and their corresponding *p*-values are reported in Table 8, alongside the estimates and standard errors calculated from the corresponding maximal model. The ideal maximal structure contains the same terms as in previous experiments, and in cases where a maximal or depleted model did not converge, additional terms were removed in the order previously specified.

**Table 7:** **Means (and Standard Errors) for Experiment 4**.

**Region**		**Critical region**	**Spillover region**
**Wh-phrase**	**Local candidate**		
**FIRST FIXATION**			
Match	Match	216 (7)	196 (6)
Match	Mismatch	229 (8)	215 (10)
Mismatch	Match	220 (8)	202 (7)
Mismatch	Mismatch	231 (9)	218 (9)
**FIRST PASS**			
Match	Match	233 (8)	387 (30)
Match	Mismatch	260 (12)	329 (22)
Mismatch	Match	241 (9)	371 (27)
Mismatch	Mismatch	259 (13)	385 (25)
**REGRESSION PATH**			
Match	Match	319 (26)	527 (59)
Match	Mismatch	352 (35)	843 (94)
Mismatch	Match	287 (23)	577 (83)
Mismatch	Mismatch	412 (46)	830 (153)
**RE-READ TIME**			
Match	Match	262 (29)	337 (59)
Match	Mismatch	468 (49)	514 (60)
Mismatch	Match	248 (24)	390 (39)
Mismatch	Mismatch	449 (54)	468 (55)

**Table 8:** **Combined ANOVA and LME results for Experiment 4**.

**Region**	**Effect**	**Estimate**	**Std error**	**χ^2^ (df)**	***p*-value**
**FIRST FIXATION**					
*Critical*	*wh*-NP	−2.12	10.12	0.04 (1)	>0.1
	local	−9.65	12.43	0.58 (1)	>0.1
	interaction	−1.01	8.14	0.03 (1)	>0.1
*Spillover*	*wh*-NP	−4.33	7.32	0.35 (1)	>0.1
	local	−15.31	9.74	2.30 (1)	>0.1
	interaction	−1.13	15.72	0.005 (1)	>0.1
**FIRST PASS**					
*Critical*	*wh*-NP	−1.66	11.87	0.02 (1)	>0.1
	local	−24.01	14.30	2.57 (1)	>0.1
	interaction	−7.99	24.89	0.10 (1)	>0.1
*Spillover*	*wh*-NP	−19.31	24.88	0.59 (1)	>0.1
	local	19.00	27.92	0.46 (1)	>0.1
	interaction	62.83	51.55	1.42 (1)	>0.1
**REGRESSION PATH**					
*Critical*	*wh*-NP	−22.48	38.62	0.33 (1)	>0.1
	local	−**81.82**	**38.71**	**3.95** (1)	**0.047**
	interaction	88.63	69.32	1.56 (1)	>0.1
*Spillover*	*wh*-NP	0.99	126.68	0.0001 (1)	>0.1
	local	−**251.72**	**96.04**	**6.19 (1)**	**0.013**
	interaction	−138.84	212.14	0.42 (1)	>0.1
**RE-READ TIME**					
*Critical*	*wh*-NP	−13.29	57.62	0.052 (1)	>0.1
	local	−**222.01**	**44.71**	**13.25 (1)**	**< 0.001**
	interaction	49.51	141.01	0.12 (1)	>0.1
*Spillover*	*wh*-NP	7.50	56.04	0.018 (1)	>0.1
	local	−**137.90**	**56.17**	**4.68** (1)	**0.031**
	interaction	−37.89	110.71	0.11 (1)	>0.1

#### 2.4.6. Results

The results of Experiment 4 reveal a significant effect in regression path duration and re-read time, consistent with Experiment 3. This main effect of local NP in the critical region reveals that gender incongruency between the local NP and the reflexive pronoun led to an increased duration than when the gender matched [regression path: β = −81.82, S.E. = 38.71, χ^2^(1) = 3.95, *p* = 0.047; re-read time: β = −222.01, S.E. = 44.71, χ^2^(1) = 13.25, *p* < 0.001].

**Figure 4:**
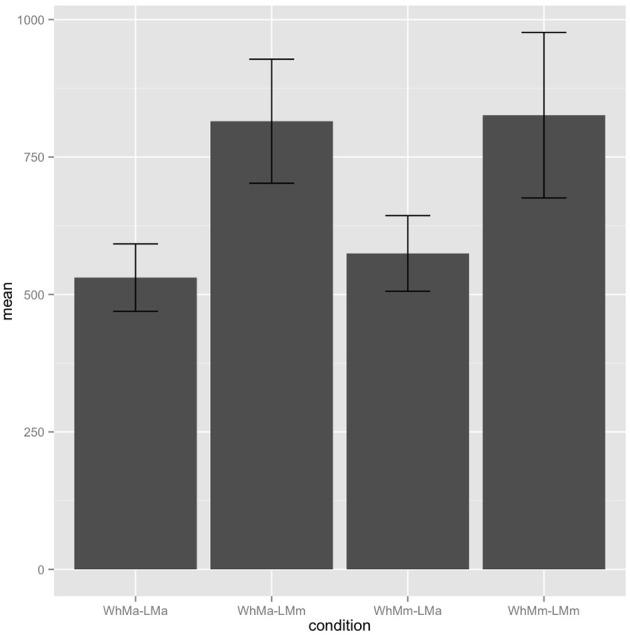
**Experiment 4 spillover region regression path durations**.

The pattern of increased durations in local mismatches is also observed in the spillover region [regression path: β = −251.72, S.E. = 96.04, χ^2^(1) = 6.19, *p* = 0.013, (Figure 4); re-read time: β = −137.90, S.E. = 56.17, χ^2^(1) = 4.68, *p* = 0.031]. This result is consistent with our claim that the parser searches a sophisticated structural representation during reflexive antecedent dependency formation. That is, the presence of an effect from the local NP supports the results of Experiment 3 in demonstrating that the parser is sensitive to the tail of the WhFGD as well as other rich, phonologically null representations in the parse tree.

## 3. Discussion

The current study sought to investigate the interaction of the resolution of two non-local dependencies, *wh*-filler-gap dependencies (WhFGD) and reflexive-antecedent dependencies (RD). In particular, we investigated the time course of the online resolution of a RD in the context of grammatically accessible and inaccessible possible antecedents. Experiments 1 and 2 examined whether the RD resolution process would target a linearly closer but grammatically illicit antecedent, or whether instead the grammatically licit tail of a WhFGD would be selected as the reflexive antecedent. Experiments 3 and 4 examined whether a possible antecedent that was both grammatically illicit and linearly more distant from a grammatically licit antecedent would influence the reflexive antecedent search. Results from these four eye-tracking text-reading experiments indicates that the RD resolution process is sensitive to grammatical structure, not local linear order.

Experiment 1 examined the time course of online reading of examples from the paradigm illustrated in (16). Crucially, such sentences are locally ambiguous; the string subsequent to the *wh*-phrase could be a coherent, grammatical utterance in which the antecedent for the reflexive would be a proper name. Globally, however, such a parse, with the proper name serving as antecedent for the reflexive, is unavailable; the only globally coherent parse is one in which the *wh*-phrase serves as the reflexive antecedent. Thus, if the search for reflexive antecedence is insensitive to syntactic structure, selecting either any feature-matching possible antecedent without regard to its structural position or the linearly closest possible antecedent, the proper name should be identified as the antecedent. Consequently, if such a theory is true, we expected to find a reading time slowdown at the reflexive if the gender of the reflexive and the proper name mismatched. Conversely, if the search for the reflexive antecedent is sensitive to grammatical configuration, we expected to see a reading time slowdown just in case the gender of the reflexive mismatched with that of the grammatically licit antecedent, the *wh*-phrase.

Results support the hypothesis that the parser is sensitive to global structural information during the reflexive antecedent search process. At or immediately after the reflexive, conditions in which the gender of the reflexive mismatched with that of the *wh*-phrase were read slower than those conditions in which the genders matched. At the spillover region the same effect of reflexive gender congruence with the *wh*-phrase was found, with the match condition read faster than the mismatch condition, in the regression path measure. We conclude that the parser attempts to form the RD with the grammatically accessible antecedent, the *wh*-phrase, and not with the grammatically inaccessible antecedent, consistent with the findings of Sturt (2003). This is despite the fact that in the configuration in question the grammatically inaccessible antecedent is linearly closer to the reflexive.

Furthermore, in Experiment 1, the string including the grammatically inaccessible antecedent and the reflexive is locally coherent if the initial *wh*-phrase is disregarded as indicated in (29-a). Theories of sentence processing where the parser builds the structure based on the information available within a linearly local span (e.g., Ferreira et al., 2002; Tabor et al., 2004; Konieczny et al., 2010) would predict that the parser could be subject to confusion in these contexts and select the linearly closer candidate antecedent, but we do not find evidence for this behavior. Given the experimental support for the existence of local coherence effects of this kind elsewhere, a possible explanation for why they do not occur in this context is that the parser operates over a representation containing the unpronounced tail of the WhFGD after the linearly local candidate antecedent. If the RD formation process operates over such a representation, local coherence effects may be blocked here because the true closest candidate antecedent is the tail of the WhFGD, i.e., there is no actual locally coherent substring in the examples because the WhFGD tail disrupts the potential local coherency (29-b).

(29)
  a. …did Mary/David expect to have injured herself/himself …
  b. …did Mary/David expect /gap/_*i*_ to have injured herself/himself …

In addition to the main effect of *wh*-phrase gender congruence found in Experiment 1, marginal interactions of *wh*-phrase gender congruence with local NP congruence were found in the regression path duration and re-read time in the critical region, such that the mismatch-mismatch conditions were read more slowly. This interaction, while not statistically significant, could be consistent with the predictions of an unconstrained cue-based model of antecedent retrieval under which the parser attempts to associate the reflexive with all possible candidate antecedents and experiences extra difficulty when no gender-congruent antecedent is found in memory.

Another interpretation for this interaction relies upon the observation that the examples used in Experiment 1 have another, less easily accessible parse in which the reflexive receives a non-argument, intensifier interpretation. Such a parse can be paraphrased with the intensifier reflexive located in another position possible for such intensifiers: *Which cowgirl did Mary herself expect to have injured due to negligence?*. It is possible that the marginal interaction with the local NP is due to the parser considering this alternative parse. Experiment 2 is an attempt to distinguish these explanations by testing configurations in which this alternative parse is unavailable.

Experiment 2 examines the reading time course of examples from the example paradigm in (21). These examples are similar to those used in Experiment 1, with the exception that the embedded clause is finite. The consequence of this change is that the examples are no longer locally ambiguous in the substring subsequent to the *wh*-phrase. However, as in Experiment 1, these examples include a grammatically accessible antecedent for the reflexive, the *wh*-phrase, and a grammatically inaccessible, but linearly closer possible antecedent, the proper name. Consequently, the gender mismatch manipulation yields the same two sets of predictions in this experiment: if the search for the reflexive antecedent is structure-insensitive, we would expect to see a gender mismatch effect on the linearly closer but grammatically inaccessible antecedent, the local NP. Conversely, if the reflexive antecedent search is sensitive to grammatical structure, we would expect to see the gender mismatch effect on the grammatically accessible but linearly further *wh*-phrase.

The results again support the hypothesis that the parser only considers the grammatically accessible *wh*-phrase when attempting to identify the reflexive antecedent. On the critical region, in regression path duration and re-read times, we saw a main effect of gender congruence with the *wh*-phrase, with the match conditions read faster than the mismatch conditions. In the spillover region, we see the same effect in first pass, regression path and re-read times. Additionally, here we failed to see any effect, even marginal, of the grammatically inaccessible antecedent. As this alternative parse is unavailable for the stimuli used in Experiment 2, this suggests that the marginal interaction with the local NP in Experiment 1 may indeed have been due to the alternative *intensifier reflexive* parse discussed above, rather than being evidence for a cue-based retrieval system experiencing difficulty in the absence of a gender-congruent candidate antecedent.

In Experiments 1 and 2, we saw that the parser considered just the grammatically licit anaphor antecedent, despite the presence of another possible antecedent intervening between the grammatically licit *wh*-phrase antecedent and the anaphor. One may wonder, however, whether these results are the result of the *wh*-phrases having a special status in working memory, or having a particularly high prominence relative to other potential antecedents (Martin and McElree, 2011). If this were the case, the results from Experiments 1 and 2 might simply be the result of this high prominence; the parser attended to the *wh*-phrase as a potential antecedent not because it was a grammatically licit antecedent and the local NP an illicit antecedent, but rather because the *wh*-phrase was the most prominent possible antecedent.

Experiments 3 and 4 were designed to test this alternative hypothesis through the examination of the reading time-course of examples from the paradigms illustrated in (25) and (27). In these examples, the *wh*-phrase is no longer a grammatically accessible antecedent for the reflexive. Instead, the local NP serves as the sole grammatically licit antecedent. Thus, if the parser considered the *wh*-phrase as an antecedent regardless of whether it is a grammatically licit antecedent, we would expect that a mismatch in gender between *wh*-phrase and anaphor would induce a slowdown in reading times.

The results of Experiments 3 and 4 do not support this alternative hypothesis. In both experiments, the conditions in which the local NP mismatched in gender with the reflexive were read slower than the conditions in which the local NP and reflexive matched in gender. In Experiment 3, this effect was found on the critical region in the first fixation, first pass, regression path, and re-read durations. In Experiment 4, the effect was found on the critical region in the regression path reading times.

The combination of Experiments 1 and 3 shows that merely having a *wh*-NP present in a sentence does not automatically cause it to be considered as the antecedent of a reflexive—that is, a general notion of the prominence of a potential antecedent, such that a *wh*-NP is checked as a potential antecedent of any dependency encountered in later processing, is insufficient to explain the observed pattern of results.

If the prominence of a potential antecedent were the source of the interference effects, we would expect to observe the same pattern in Experiments 1 and 3. But instead the observed pattern is that, when the tail of the WhFGD intervenes between the linearly local embedded subject NP and the reflexive, the *wh*-NP's gender congruency with the reflexive modulates the presence or absence of gender mismatch effects, whereas when the the *wh*-NP is not associated with a tail intervening between the subject and reflexive, the linearly local embedded subject NP's gender congruency with the reflexive modulates the presence or absence of gender mismatch effects. Thus, while it is plausibly true that *wh*-NPs are highly “prominent” candidate antecedents, the parser still appears to be guided by syntactic structure in the course of reflexive resolution and is not “confused” by the presence of an irrelevant *wh*-NP.

The principal significance of this set of findings is to provide evidence for quite sophisticated structure-sensitivity on the part of the antecedent retrieval system. Whatever mechanism subserves reflexive antecedent retrieval, whether cue-based or otherwise, must be able to exhibit online sensitivity to fine-grained syntactic structure of at least two kinds. First, it must be able to respect the clausemate condition on anaphora: that reflexives are unable to find their antecedent outside of their immediate clause. Second, it must be sensitive to the presence and location of WhFGD tails: the presence of a WhFGD must be visible to the antecedent retrieval system, whether in the form of reactivation of a previously processed NP upon gap-detection or via the positing of gaps/dependency tails as candidate antecedents in the representation over which reflexive antecedent retrieval operates. For this reason, this study constitutes evidence against unrestricted versions of cue-based retrieval, and in favor of models like that in Dillon et al. (2013) that constrain the antecedent retrieval process to respect syntactic structure.

Why might the reflexive antecedent retrieval system fail to experience interference from grammatically inaccessible antecedents in WhFGD contexts, while showing evidence of such interference in reflexive antecedent retrieval mediated by the related long-distance dependencies of raising and control studied in Kwon and Sturt (2015) and Sturt and Kwon (2015)? We speculate that the active nature of the WhFGD formation process may provide an explanation for this difference. Encountering a *wh*-NP triggers the parser to initiate an active search for its corresponding gap site, while control and raising dependencies cannot be identified until later in a sentence. If active search behavior on the part of the parser involves positing a WhFGD dependency tail within the local domain of the reflexive, this element may be an accessible retrieval candidate for a syntactically constrained antecedent retrieval system. Future research could address this question by investigating reflexive antecedent retrieval in the context of other long-distance dependencies whose leftmost element is a strong cue to the existence of the long-distance dependency, perhaps topicalization.

## 4. Conclusion

In this study we have investigated whether the process of reflexive-antecedent resolution is sensitive, in on-line measures, to the presence of a WhFGD dependency whose tail is the grammatically licit antecedent of the reflexive. The fact that Experiments 1 and 2 found gender mismatch effects between the *wh*-NP and the reflexive, and not between a linearly local NP and the reflexive, strongly supports the position that the tail of a WhFGD can be accessed rapidly online as a candidate antecedent in reflexive antecedent search.

This effect of the *wh*-NP is not compatible with an account where *wh*-NPs are simply highly prominent candidate antecedents regardless of the grammatical possibility of such an antecedent-reflexive relationship because, if this were the explanation for the effect of gender mismatch between *wh*-NP and reflexive in Experiments 1 and 2, Experiments 3 and 4 should have shown the same pattern. Instead, in Experiments 3 and 4, gender mismatch effects of the linearly local non-*wh*-NP and the reflexive were observed, consistent with an account on which the parser's reflexive antecedent search is grammatically guided. In general, then, we conclude that the parser's reflexive antecedent search is rapidly sensitive to such fine-grained syntactic details as the presence and location of a WhFGD. We take this to be evidence that, whatever mechanism is implicated in reflexive antecedent retrieval, it must be able to exhibit online sensitivity to the binding constraints and to treat the tail of a WhFGD as a potential candidate antecedent.

### Conflict of interest statement

The authors declare that the research was conducted in the absence of any commercial or financial relationships that could be construed as a potential conflict of interest.

## Footnotes

^1^Here and throughout, subscript indices are used to indicate possible and impossible coreference relations: NPs bearing the same subscript index indicate an interpretation under which these NPs refer to the same entity, and impossible interpretations [e.g., *himself* = *Jane* in (1)] are prefixed with an asterisk.

^2^Whether *position* here is defined structurally, or in terms of verbal selection frames, or at the level of predicate-argument relations, is immaterial at this early point in the discussion.

^3^While (Stowe, 1986) observed FGE-related slowdown only in the position of non-subjects, not of subjects, since then, other researchers, for example Lee (2004), have found that subject-FGEs do appear under slightly different experimental settings.

^4^C-command (Reinhart, 1976) is a notion of relative syntactic prominence; formally, in a tree structure, α c-commands β iff α does not dominate β and the node immediately dominating α also dominates β. For our purposes here, it suffices that subjects c-command their associated verb phrases and all the contents of their associated verb phrases, including subordinate clauses. Note also that additional NPs contained inside an NP subject do not c-command anything out of the NP subject, as in (7-b) below.

^5^Note that a consequence of this experimental manipulation is that conditions in which the only grammatically-accessible antecedent for the reflexive mismatches it in gender are prima facie ungrammatical because, for example, *The cowgirl injured himself* or *Steven injured herself* may not be grammatical reflexive-antecedent dependencies. However, it is arguable whether examples like *The cowgirl injured himself* or *Steven injured herself* are genuinely ungrammatical, rather than simply unacceptable in the majority of contexts: it is not unimaginable that a woman might be named *Steven*, merely very unexpected, and likewise in the context of a costume party, the individual picked out by the referring expression *the cowgirl* could conceivably be male.

^6^Modulo dispreferred intensifier readings of the reflexive which will be addressed in the discussion below.

^7^For example: *lmer*(*rt* ∽ *wh*^*^*lc* + *ord* + (1 + *wh*^*^*lc* + *ord*|*subj*) + (1 + *wh*^*^*lc* + *ord*|*item*), *data* = *data*), where *rt* is the reading time, the predictors *wh* and *lc* are the gender match/mismatch of the *wh*-NP and the local candidate antecedent NP, respectively, and *ord* is the presentation order.

^8^Thanks to Dave Kush (*personal communication*) for pointing out the possibility of this reading for the stimuli in Experiment 1.

^9^The smaller number of participants in this experiment is due to accidental exclusion of the target stimuli during compiling for experimental presentation in half of the presentation orders. Fortunately, the conditions remain properly counterbalanced.
