# Supplementary material for: Wh-filler-gap dependency formation guides reflexive antecedent search
Source: Front Psychol. 2015 Oct 9;6:1504. doi: 10.3389/fpsyg.2015.01504 (PMC4599314; doi:10.3389/fpsyg.2015.01504)
Supplement: Supplementary file 1 [file DataSheet1.PDF]

1 APPENDIX

1.1 EXPERIMENT 1 STIMULI

1. a. Which saleswoman did Margaret presume to have excused herself for unimportant reasons?
   b. Which saleswoman did Margaret presume to have excused himself for unimportant reasons?
   c. Which saleswoman did Edward presume to have excused herself for unimportant reasons?
   d. Which saleswoman did Edward presume to have excused himself for unimportant reasons?

2. a. Which Englishwoman did Cynthia proclaim to have restored herself to his former honor?
   b. Which Englishwoman did Cynthia proclaim to have restored himself to his former honor?
   c. Which Englishwoman did Henry proclaim to have restored herself to his former honor?
   d. Which Englishwoman did Henry proclaim to have restored himself to his former honor?

3. a. Which woman did Donna remember to have groomed herself with great care?
   b. Which woman did Donna remember to have groomed himself with great care?
   c. Which woman did Scott remember to have groomed herself with great care?
   d. Which woman did Scott remember to have groomed himself with great care?

4. a. Which policewoman did Dorothy report to have disgraced herself by embezzling funds?
   b. Which policewoman did Dorothy report to have disgraced himself by embezzling funds?
   c. Which policewoman did Larry report to have disgraced herself by embezzling funds?
   d. Which policewoman did Larry report to have disgraced himself by embezzling funds?

5. a. Which spokeswoman did Brenda disclose to have exposed herself to potential conflicts of interest?
   b. Which spokeswoman did Brenda disclose to have exposed himself to potential conflicts of interest?
   c. Which spokeswoman did Jose disclose to have exposed herself to potential conflicts of interest?
   d. Which spokeswoman did Jose disclose to have exposed himself to potential conflicts of interest?

6. a. Which cowgirl did Anna figure to have injured herself due to negligence?
   b. Which cowgirl did Anna figure to have injured himself due to negligence?
   c. Which cowgirl did Steven figure to have injured herself due to negligence?
   d. Which cowgirl did Steven figure to have injured himself due to negligence?

7. a. Which lady did Pamela discover to have been fooling herself about the presidential election?
   b. Which lady did Pamela discover to have been fooling himself about the presidential election?
   c. Which lady did Robert discover to have been fooling herself about the presidential election?
   d. Which lady did Robert discover to have been fooling himself about the presidential election?

8. a. Which aunt did Carolyn reveal to have affiliated herself with a crime syndicate?
   b. Which aunt did Carolyn reveal to have affiliated himself with a crime syndicate?
   c. Which aunt did Kevin reveal to have affiliated herself with a crime syndicate?
   d. Which aunt did Kevin reveal to have affiliated himself with a crime syndicate?

9. a. Which weathergirl did Virginia state to have burned herself after being outside for too long?
   b. Which weathergirl did Virginia state to have burned himself after being outside for too long?
   c. Which weathergirl did Kenneth state to have burned herself after being outside for too long?
   d. Which weathergirl did Kenneth state to have burned himself after being outside for too long?

10. a. Which wife did Jessica verify to have taught herself how to swim?
    b. Which wife did Jessica verify to have taught himself how to swim?
    c. Which wife did Paul verify to have taught herself how to swim?
    d. Which wife did Paul verify to have taught himself how to swim?

11. a. Which actress did Lisa imagine to have motivated herself to stay up all night working?
    b. Which actress did Lisa imagine to have motivated himself to stay up all night working?

c. Which actress did James imagine to have motivated herself to stay up all night working?
d. Which actress did James imagine to have motivated himself to stay up all night working?

12. a. Which grandma did Marie demonstrate to have been protecting herself enough from bears?
b. Which grandma did Marie demonstrate to have been protecting himself enough from bears?
c. Which grandma did Joshua demonstrate to have been protecting herself enough from bears?
d. Which grandma did Joshua demonstrate to have been protecting himself enough from bears?

13. a. Which princess did Stephanie believe to have prepared herself a forged certification document?
b. Which princess did Stephanie believe to have prepared himself a forged certification document?
c. Which princess did Jeffrey believe to have prepared herself a forged certification document?
d. Which princess did Jeffrey believe to have prepared himself a forged certification document?

14. a. Which queen did Martha know to have made herself a cocktail without paying for it?
b. Which queen did Martha know to have made himself a cocktail without paying for it?
c. Which queen did George know to have made herself a cocktail without paying for it?
d. Which queen did George know to have made himself a cocktail without paying for it?

15. a. Which man did Douglas recognize to have doped himself for the whole season?
b. Which man did Douglas recognize to have doped herself for the whole season?
c. Which man did Rebecca recognize to have doped himself for the whole season?
d. Which man did Rebecca recognize to have doped herself for the whole season?

16. a. Which guy did Harold observe to have familiarized himself with the directions?
b. Which guy did Harold observe to have familiarized herself with the directions?
c. Which guy did Sharon observe to have familiarized himself with the directions?
d. Which guy did Sharon observe to have familiarized herself with the directions?

17. a. Which boy did Joseph judge to have proven himself in the regional tournament?
b. Which boy did Joseph judge to have proven herself in the regional tournament?

c. Which boy did Sandra judge to have proven himself in the regional tournament?
d. Which boy did Sandra judge to have proven herself in the regional tournament?

18. a. Which brother did Andrew note to have persuaded himself to purchase a new talisman?
b. Which brother did Andrew note to have persuaded herself to purchase a new talisman?
c. Which brother did Elizabeth note to have persuaded himself to purchase a new talisman?
d. Which brother did Elizabeth note to have persuaded herself to purchase a new talisman?

19. a. Which schoolboy did Stephen guess to have injured himself in the yard?
b. Which schoolboy did Stephen guess to have injured herself in the yard?
c. Which schoolboy did Janet guess to have injured himself in the yard?
d. Which schoolboy did Janet guess to have injured herself in the yard?

20. a. Which businessman did Brian judge to have been flattering himself during the reception?
b. Which businessman did Brian judge to have been flattering herself during the reception?
c. Which businessman did Amanda judge to have been flattering himself during the reception?
d. Which businessman did Amanda judge to have been flattering herself during the reception?

21. a. Which groomsman did William believe to have overindulged himself at the shopping center?
b. Which groomsman did William believe to have overindulged herself at the shopping center?
c. Which groomsman did Catherine believe to have overindulged himself at the shopping center?
d. Which groomsman did Catherine believe to have overindulged herself at the shopping center?

22. a. Which choirboy did Frank take to have humiliated himself during the craft convention?
b. Which choirboy did Frank take to have humiliated herself during the craft convention?
c. Which choirboy did Barbara take to have humiliated himself during the craft convention?
d. Which choirboy did Barbara take to have humiliated herself during the craft convention?

23. a. Which bachelor did Timothy report to have saved himself from the burning building?
b. Which bachelor did Timothy report to have saved herself from the burning building?
c. Which bachelor did Mary report to have saved himself from the burning building?
d. Which bachelor did Mary report to have saved herself from the burning building?

24. a. Which prince did Thomas believe to have exposed himself to a dangerous neurological toxin?
    b. Which prince did Thomas believe to have exposed herself to a dangerous neurological toxin?
    c. Which prince did Karen believe to have exposed himself to a dangerous neurological toxin?
    d. Which prince did Karen believe to have exposed herself to a dangerous neurological toxin?

1.2 EXPERIMENT 2 STIMULI

1. a. Which saleswoman did Margaret presume had excused herself for unimportant reasons?
   b. Which saleswoman did Margaret presume had excused himself for unimportant reasons?
   c. Which saleswoman did Edward presume had excused herself for unimportant reasons?
   d. Which saleswoman did Edward presume had excused himself for unimportant reasons?

2. a. Which Englishwoman did Cynthia proclaim had restored herself to their former honor?
   b. Which Englishwoman did Cynthia proclaim had restored himself to their former honor?
   c. Which Englishwoman did Henry proclaim had restored herself to their former honor?
   d. Which Englishwoman did Henry proclaim had restored himself to their former honor?

3. a. Which woman did Donna remember had groomed herself with great care?
   b. Which woman did Donna remember had groomed himself with great care?
   c. Which woman did Scott remember had groomed herself with great care?
   d. Which woman did Scott remember had groomed himself with great care?

4. a. Which policewoman did Dorothy report had disgraced herself by embezzling funds?
   b. Which policewoman did Dorothy report had disgraced himself by embezzling funds?
   c. Which policewoman did Larry report had disgraced herself by embezzling funds?
   d. Which policewoman did Larry report had disgraced himself by embezzling funds?

5. a. Which spokeswoman did Brenda disclose had exposed herself to potential conflicts of interest?
   b. Which spokeswoman did Brenda disclose had exposed himself to potential conflicts of interest?
   c. Which spokeswoman did Jose disclose had exposed herself to potential conflicts of interest?
   d. Which spokeswoman did Jose disclose had exposed himself to potential conflicts of interest?

6. a. Which cowgirl did Anna figure had injured herself due to negligence?

b. Which cowgirl did Anna figure had injured himself due to negligence?
c. Which cowgirl did Steven figure had injured herself due to negligence?
d. Which cowgirl did Steven figure had injured himself due to negligence?

7. a. Which lady did Pamela discover had been fooling herself about the dangers of processed foods?
b. Which lady did Pamela discover had been fooling himself about the dangers of processed foods?
c. Which lady did Robert discover had been fooling herself about the dangers of processed foods?
d. Which lady did Robert discover had been fooling himself about the dangers of processed foods?

8. a. Which aunt did Carolyn reveal had affiliated herself with a crime syndicate?
b. Which aunt did Carolyn reveal had affiliated himself with a crime syndicate?
c. Which aunt did Kevin reveal had affiliated herself with a crime syndicate?
d. Which aunt did Kevin reveal had affiliated himself with a crime syndicate?

9. a. Which weathergirl did Kathleen state had injured herself after neglecting the research protocols?
b. Which weathergirl did Kathleen state had injured himself after neglecting the research protocols?
c. Which weathergirl did Kenneth state had injured herself after neglecting the research protocols?
d. Which weathergirl did Kenneth state had injured himself after neglecting the research protocols?

10. a. Which wife did Jessica verify had allowed herself to succumb to the temptation to have an affair?
b. Which wife did Jessica verify had allowed himself to succumb to the temptation to have an affair?
c. Which wife did Paul verify had allowed herself to succumb to the temptation to have an affair?
d. Which wife did Paul verify had allowed himself to succumb to the temptation to have an affair?

11. a. Which actress did Lisa imagine had motivated herself to stay up all night working?
b. Which actress did Lisa imagine had motivated himself to stay up all night working?
c. Which actress did James imagine had motivated herself to stay up all night working?
d. Which actress did James imagine had motivated himself to stay up all night working?

12. a. Which grandma did Marie demonstrate had been protecting herself enough from heart disease?
b. Which grandma did Marie demonstrate had been protecting himself enough from heart disease?

c. Which grandma did Joshua demonstrate had been protecting herself enough from heart disease?
d. Which grandma did Joshua demonstrate had been protecting himself enough from heart disease?

13. a. Which princess did Stephanie believe had prepared herself a forged certification document?
b. Which princess did Stephanie believe had prepared himself a forged certification document?
c. Which princess did Jeffrey believe had prepared herself a forged certification document?
d. Which princess did Jeffrey believe had prepared himself a forged certification document?

14. a. Which queen did Martha know had involved herself in political corruption?
b. Which queen did Martha know had involved himself in political corruption?
c. Which queen did George know had involved herself in political corruption?
d. Which queen did George know had involved himself in political corruption?

15. a. Which man did Douglas recognize had doped himself for the whole season?
b. Which man did Douglas recognize had doped herself for the whole season?
c. Which man did Rebecca recognize had doped himself for the whole season?
d. Which man did Rebecca recognize had doped herself for the whole season?

16. a. Which guy did Harold observe had familiarized himself with the directions?
b. Which guy did Harold observe had familiarized herself with the directions?
c. Which guy did Sharon observe had familiarized himself with the directions?
d. Which guy did Sharon observe had familiarized herself with the directions?

17. a. Which boy did Joseph judge had proven himself in the regional tournament?
b. Which boy did Joseph judge had proven herself in the regional tournament?
c. Which boy did Sandra judge had proven himself in the regional tournament?
d. Which boy did Sandra judge had proven herself in the regional tournament?

18. a. Which brother did Andrew note had persuaded himself to buy a new laptop?
b. Which brother did Andrew note had persuaded herself to buy a new laptop?

c. Which brother did Elizabeth note had persuaded himself to buy a new laptop?
d. Which brother did Elizabeth note had persuaded herself to buy a new laptop?

19. a. Which schoolboy did Stephen guess had injured himself in the yard?
b. Which schoolboy did Stephen guess had injured herself in the yard?
c. Which schoolboy did Janet guess had injured himself in the yard?
d. Which schoolboy did Janet guess had injured herself in the yard?

20. a. Which businessman did Brian judge had been flattering himself during the reception?
b. Which businessman did Brian judge had been flattering herself during the reception?
c. Which businessman did Amanda judge had been flattering himself during the reception?
d. Which businessman did Amanda judge had been flattering herself during the reception?

21. a. Which groomsman did William believe had overindulged himself at the shopping center?
b. Which groomsman did William believe had overindulged herself at the shopping center?
c. Which groomsman did Catherine believe had overindulged himself at the shopping center?
d. Which groomsman did Catherine believe had overindulged herself at the shopping center?

22. a. Which choirboy did Frank think had humiliated himself during the craft convention?
b. Which choirboy did Frank think had humiliated herself during the craft convention?
c. Which choirboy did Barbara think had humiliated himself during the craft convention?
d. Which choirboy did Barbara think had humiliated herself during the craft convention?

23. a. Which bachelor did Timothy report had saved himself from the burning building?
b. Which bachelor did Timothy report had saved herself from the burning building?
c. Which bachelor did Mary report had saved himself from the burning building?
d. Which bachelor did Mary report had saved herself from the burning building?

24. a. Which prince did Thomas believe had exposed himself to a dangerous neurological toxin?
b. Which prince did Thomas believe had exposed herself to a dangerous neurological toxin?
c. Which prince did Karen believe had exposed himself to a dangerous neurological toxin?
d. Which prince did Karen believe had exposed herself to a dangerous neurological toxin?

1.3 EXPERIMENT 3 STIMULI

1. a. Which saleswoman presumed Margaret to have excused herself for unimportant reasons?
   b. Which saleswoman presumed Margaret to have excused himself for unimportant reasons?
   c. Which saleswoman presumed Edward to have excused herself for unimportant reasons?
   d. Which saleswoman presumed Edward to have excused himself for unimportant reasons?

2. a. Which Englishwoman proclaimed Cynthia to have restored herself to their former honor?
   b. Which Englishwoman proclaimed Cynthia to have restored himself to their former honor?
   c. Which Englishwoman proclaimed Henry to have restored herself to their former honor?
   d. Which Englishwoman proclaimed Henry to have restored himself to their former honor?

3. a. Which woman remembered Donna to have groomed herself with great care?
   b. Which woman remembered Donna to have groomed himself with great care?
   c. Which woman remembered Scott to have groomed herself with great care?
   d. Which woman remembered Scott to have groomed himself with great care?

4. a. Which policewoman reported Dorothy to have disgraced herself by embezzling funds?
   b. Which policewoman reported Dorothy to have disgraced himself by embezzling funds?
   c. Which policewoman reported Larry to have disgraced herself by embezzling funds?
   d. Which policewoman reported Larry to have disgraced himself by embezzling funds?

5. a. Which spokeswoman disclosed Brenda to have exposed herself to potential conflicts of interest?
   b. Which spokeswoman disclosed Brenda to have exposed himself to potential conflicts of interest?
   c. Which spokeswoman disclosed Jose to have exposed herself to potential conflicts of interest?
   d. Which spokeswoman disclosed Jose to have exposed himself to potential conflicts of interest?

6. a. Which cowgirl figured Anna to have injured herself due to negligence?
   b. Which cowgirl figured Anna to have injured himself due to negligence?
   c. Which cowgirl figured Steven to have injured herself due to negligence?
   d. Which cowgirl figured Steven to have injured himself due to negligence?

7. a. Which lady discovered Pamela to have been fooling herself about the dangers of processed foods?
   b. Which lady discovered Pamela to have been fooling himself about the dangers of processed foods?
   c. Which lady discovered Robert to have been fooling herself about the dangers of processed foods?

d. Which lady discovered Robert to have been fooling himself about the dangers of processed foods?

8. a. Which aunt revealed Carolyn to have affiliated herself with a crime syndicate?
b. Which aunt revealed Carolyn to have affiliated himself with a crime syndicate?
c. Which aunt revealed Kevin to have affiliated herself with a crime syndicate?
d. Which aunt revealed Kevin to have affiliated himself with a crime syndicate?

9. a. Which weathergirl stated Kathleen to have injured herself after neglecting the research protocols?
b. Which weathergirl stated Kathleen to have injured himself after neglecting the research protocols?
c. Which weathergirl stated Kenneth to have injured herself after neglecting the research protocols?
d. Which weathergirl stated Kenneth to have injured himself after neglecting the research protocols?

10. a. Which wife verified Jessica to have allowed herself to succumb to the temptation to have an affair?
b. Which wife verified Jessica to have allowed himself to succumb to the temptation to have an affair?
c. Which wife verified Paul to have allowed herself to succumb to the temptation to have an affair?
d. Which wife verified Paul to have allowed himself to succumb to the temptation to have an affair?

11. a. Which actress imagined Lisa to have motivated herself to stay up all night working?
b. Which actress imagined Lisa to have motivated himself to stay up all night working?
c. Which actress imagined James to have motivated herself to stay up all night working?
d. Which actress imagined James to have motivated himself to stay up all night working?

12. a. Which grandma demonstrated Marie to have been protecting herself enough from heart disease?
b. Which grandma demonstrated Marie to have been protecting himself enough from heart disease?
c. Which grandma demonstrated Joshua to have been protecting herself enough from heart disease?
d. Which grandma demonstrated Joshua to have been protecting himself enough from heart disease?

13. a. Which princess believed Stephanie to have prepared herself a forged certification document?
b. Which princess believed Stephanie to have prepared himself a forged certification document?
c. Which princess believed Jeffrey to have prepared herself a forged certification document?

d. Which princess believed Jeffrey to have prepared himself a forged certification document?

14.
a. Which queen knew Martha to have involved herself in political corruption?
b. Which queen knew Martha to have involved himself in political corruption?
c. Which queen knew George to have involved herself in political corruption?
d. Which queen knew George to have involved himself in political corruption?

15.
a. Which man recognized Douglas to have doped himself for the whole season?
b. Which man recognized Douglas to have doped herself for the whole season?
c. Which man recognized Rebecca to have doped himself for the whole season?
d. Which man recognized Rebecca to have doped herself for the whole season?

16.
a. Which guy observed Harold to have familiarized himself with the directions?
b. Which guy observed Harold to have familiarized herself with the directions?
c. Which guy observed Sharon to have familiarized himself with the directions?
d. Which guy observed Sharon to have familiarized herself with the directions?

17.
a. Which boy judged Joseph to have proven himself in the regional tournament?
b. Which boy judged Joseph to have proven herself in the regional tournament?
c. Which boy judged Sandra to have proven himself in the regional tournament?
d. Which boy judged Sandra to have proven herself in the regional tournament?

18.
a. Which brother noted Andrew to have persuaded himself to buy a new laptop?
b. Which brother noted Andrew to have persuaded herself to buy a new laptop?
c. Which brother noted Elizabeth to have persuaded himself to buy a new laptop?
d. Which brother noted Elizabeth to have persuaded herself to buy a new laptop?

19.
a. Which schoolboy guessed Stephen to have injured himself in the yard?
b. Which schoolboy guessed Stephen to have injured herself in the yard?
c. Which schoolboy guessed Janet to have injured himself in the yard?
d. Which schoolboy guessed Janet to have injured herself in the yard?

20. a. Which businessman judged Brian to have been flattering himself during the reception?
    b. Which businessman judged Brian to have been flattering herself during the reception?
    c. Which businessman judged Amanda to have been flattering himself during the reception?
    d. Which businessman judged Amanda to have been flattering herself during the reception?

21. a. Which groomsman believed William to have overindulged himself at the shopping center?
    b. Which groomsman believed William to have overindulged herself at the shopping center?
    c. Which groomsman believed Catherine to have overindulged himself at the shopping center?
    d. Which groomsman believed Catherine to have overindulged herself at the shopping center?

22. a. Which choirboy took Frank to have humiliated himself during the craft convention?
    b. Which choirboy took Frank to have humiliated herself during the craft convention?
    c. Which choirboy took Barbara to have humiliated himself during the craft convention?
    d. Which choirboy took Barbara to have humiliated herself during the craft convention?

23. a. Which bachelor reported Timothy to have saved himself from the burning building?
    b. Which bachelor reported Timothy to have saved herself from the burning building?
    c. Which bachelor reported Mary to have saved himself from the burning building?
    d. Which bachelor reported Mary to have saved herself from the burning building?

24. a. Which prince believed Thomas to have exposed himself to a dangerous neurological toxin?
    b. Which prince believed Thomas to have exposed herself to a dangerous neurological toxin?
    c. Which prince believed Karen to have exposed himself to a dangerous neurological toxin?
    d. Which prince believed Karen to have exposed herself to a dangerous neurological toxin?

1.4 EXPERIMENT 4 STIMULI

1. a. Which saleswoman presumed Margaret had excused herself for unimportant reasons?
   b. Which saleswoman presumed Margaret had excused himself for unimportant reasons?
   c. Which saleswoman presumed Edward had excused herself for unimportant reasons?

d. Which saleswoman presumed Edward had excused himself for unimportant reasons?

2. a. Which Englishwoman proclaimed Cynthia had restored herself to their former honor?
   b. Which Englishwoman proclaimed Cynthia had restored himself to their former honor?
   c. Which Englishwoman proclaimed Henry had restored herself to their former honor?
   d. Which Englishwoman proclaimed Henry had restored himself to their former honor?

3. a. Which woman remembered Donna had groomed herself with great care?
   b. Which woman remembered Donna had groomed himself with great care?
   c. Which woman remembered Scott had groomed herself with great care?
   d. Which woman remembered Scott had groomed himself with great care?

4. a. Which policewoman reported Dorothy had disgraced herself by embezzling funds?
   b. Which policewoman reported Dorothy had disgraced himself by embezzling funds?
   c. Which policewoman reported Larry had disgraced herself by embezzling funds?
   d. Which policewoman reported Larry had disgraced himself by embezzling funds?

5. a. Which spokeswoman disclosed Brenda had exposed herself to potential conflicts of interest?
   b. Which spokeswoman disclosed Brenda had exposed himself to potential conflicts of interest?
   c. Which spokeswoman disclosed Jose had exposed herself to potential conflicts of interest?
   d. Which spokeswoman disclosed Jose had exposed himself to potential conflicts of interest?

6. a. Which cowgirl figured Anna had injured herself due to negligence?
   b. Which cowgirl figured Anna had injured himself due to negligence?
   c. Which cowgirl figured Steven had injured herself due to negligence?
   d. Which cowgirl figured Steven had injured himself due to negligence?

7. a. Which lady discovered Pamela had been fooling herself about the dangers of processed foods?
   b. Which lady discovered Pamela had been fooling himself about the dangers of processed foods?
   c. Which lady discovered Robert had been fooling herself about the dangers of processed foods?
   d. Which lady discovered Robert had been fooling himself about the dangers of processed foods?

8. a. Which aunt revealed Carolyn had affiliated herself with a crime syndicate?
   b. Which aunt revealed Carolyn had affiliated himself with a crime syndicate?

c. Which aunt revealed Kevin had affiliated herself with a crime syndicate?
d. Which aunt revealed Kevin had affiliated himself with a crime syndicate?

9. a. Which weathergirl stated Kathleen had injured herself after neglecting the research protocols?
b. Which weathergirl stated Kathleen had injured himself after neglecting the research protocols?
c. Which weathergirl stated Kenneth had injured herself after neglecting the research protocols?
d. Which weathergirl stated Kenneth had injured himself after neglecting the research protocols?

10. a. Which wife verified Jessica had allowed herself to succumb to the temptation to have an affair?
b. Which wife verified Jessica had allowed himself to succumb to the temptation to have an affair?
c. Which wife verified Paul had allowed herself to succumb to the temptation to have an affair?
d. Which wife verified Paul had allowed himself to succumb to the temptation to have an affair?

11. a. Which actress imagined Lisa had motivated herself to stay up all night working?
b. Which actress imagined Lisa had motivated himself to stay up all night working?
c. Which actress imagined James had motivated herself to stay up all night working?
d. Which actress imagined James had motivated himself to stay up all night working?

12. a. Which grandma demonstrated Marie had been protecting herself enough from heart disease?
b. Which grandma demonstrated Marie had been protecting himself enough from heart disease?
c. Which grandma demonstrated Joshua had been protecting herself enough from heart disease?
d. Which grandma demonstrated Joshua had been protecting himself enough from heart disease?

13. a. Which princess believed Stephanie had prepared herself a forged certification document?
b. Which princess believed Stephanie had prepared himself a forged certification document?
c. Which princess believed Jeffrey had prepared herself a forged certification document?
d. Which princess believed Jeffrey had prepared himself a forged certification document?

14. a. Which queen knew Martha had involved herself in political corruption?
b. Which queen knew Martha had involved himself in political corruption?

c. Which queen knew George had involved herself in political corruption?
d. Which queen knew George had involved himself in political corruption?

15. a. Which man recognized Douglas had doped himself for the whole season?
b. Which man recognized Douglas had doped herself for the whole season?
c. Which man recognized Rebecca had doped himself for the whole season?
d. Which man recognized Rebecca had doped herself for the whole season?

16. a. Which guy observed Harold had familiarized himself with the directions?
b. Which guy observed Harold had familiarized herself with the directions?
c. Which guy observed Sharon had familiarized himself with the directions?
d. Which guy observed Sharon had familiarized herself with the directions?

17. a. Which boy judged Joseph had proven himself in the regional tournament?
b. Which boy judged Joseph had proven herself in the regional tournament?
c. Which boy judged Sandra had proven himself in the regional tournament?
d. Which boy judged Sandra had proven herself in the regional tournament?

18. a. Which brother noted Andrew had persuaded himself to buy a new laptop?
b. Which brother noted Andrew had persuaded herself to buy a new laptop?
c. Which brother noted Elizabeth had persuaded himself to buy a new laptop?
d. Which brother noted Elizabeth had persuaded herself to buy a new laptop?

19. a. Which schoolboy guessed Stephen had injured himself in the yard?
b. Which schoolboy guessed Stephen had injured herself in the yard?
c. Which schoolboy guessed Janet had injured himself in the yard?
d. Which schoolboy guessed Janet had injured herself in the yard?

20. a. Which businessman judged Brian had been flattering himself during the reception?
b. Which businessman judged Brian had been flattering herself during the reception?
c. Which businessman judged Amanda had been flattering himself during the reception?
d. Which businessman judged Amanda had been flattering herself during the reception?

21. a. Which groomsman believed William had overindulged himself at the shopping center?
b. Which groomsman believed William had overindulged herself at the shopping center?
c. Which groomsman believed Catherine had overindulged himself at the shopping center?
d. Which groomsman believed Catherine had overindulged herself at the shopping center?

22. a. Which choirboy thought Frank had humiliated himself during the craft convention?
b. Which choirboy thought Frank had humiliated herself during the craft convention?
c. Which choirboy thought Barbara had humiliated himself during the craft convention?
d. Which choirboy thought Barbara had humiliated herself during the craft convention?

23. a. Which bachelor reported Timothy had saved himself from the burning building?
b. Which bachelor reported Timothy had saved herself from the burning building?
c. Which bachelor reported Mary had saved himself from the burning building?
d. Which bachelor reported Mary had saved herself from the burning building?

24. a. Which prince believed Thomas had exposed himself to a dangerous neurological toxin?
b. Which prince believed Thomas had exposed herself to a dangerous neurological toxin?
c. Which prince believed Karen had exposed himself to a dangerous neurological toxin?
d. Which prince believed Karen had exposed herself to a dangerous neurological toxin?
